# Supplementary material for: Azoles activate type I and type II programmed cell death pathways in crop pathogenic fungi
Source: Nat Commun. 2024 May 31;15:4357. doi: 10.1038/s41467-024-48157-9 (PMC11143370; doi:10.1038/s41467-024-48157-9)
Supplement: Supplementary file 1 — Supplementary Information [file 41467_2024_48157_MOESM1_ESM.pdf]

Supplementary Information

Title: Azoles activate type I and type II programmed cell death pathways in crop pathogenic fungi

Authors: Schuster, M., Kilaru, S., Steinberg, G.

Contents

Supplementary Figures and Figure Legends

Supp. Figure 1: Effects of azoles on the plasma membrane in *Z. tritici*.

Supp. Figure 2: Effect of azoles on plate growth of *Z. tritici* and *M. oryzae.*

Supp. Figure 3: Effect of high concentration of azoles on growth and mortality in *Z. tritici*.

Supp. Figure 4: Azoles induce incomplete septation.

Supp. Figure 5: Localisation of an Imp2 homologue in epoxiconazole-treated *Z. tritici* cells.

Supp. Figure 6: Azoles hyperpolarise mitochondria and increase cellular ATP.

Supp. Figure 7: Mitochondria organization, hyper-polarization and ROS development in azole-treated *Z. tritici* cells.

Supp. Figure 8: Azoles induce mROS-dependent apoptosis.

Supp. Figure 9: Indications of apoptosis in epoxiconazole-treated *Z. tritici* cells.

Supp. Figure 10: Azoles kill *Z. tritici* cells by inducing apoptosis and autophagy.

Supp. Figure 11: Data supporting results around azole-induced autophagy in *Z. tritici* cells.

Supp. Figure 12: MoA of azoles at higher concentrations in *Z. tritici*.

Supp. Figure 13: Activity of epoxiconazole in *Z. tritici* during early plant infection.

Supp. Figure 14: The rice blast pathogen *M. oryzae* shares the azole MoA with *Z. tritici*.

Supp. Figure 15: Fungicidal activity of azoles in *M. oryzae*.

Supp. Figure 16: Ultrastructure of septae in azole-treated cells of *M. oryzae* hyphae.

Supp. Figure 17: Targets of inhibitors of the ergosterol biosynthesis pathway.

Supp. Figure 18: Ergosterol biosynthesis inhibitors have a common MoA.

Supp. Figure 19: Effect of external ergosterol in epoxiconazole-treated *Z. tritici* cells.

Supp. Figure 20: The MoA of ergosterol biosynthesis inhibitors

Supplementary Tables

Supp. Table 1: Genotypes of strains used in this study.

Supp. Table 2: Plasmids used in this study.

Supp. Table 3: Experimental strain usage.

Supp. Table 4: Reporter genes to study the MoA of azoles *in planta*.

Supp. Table 5: Primers used in this study.

Supplementary References

Supplementary Figures and Figure Legends

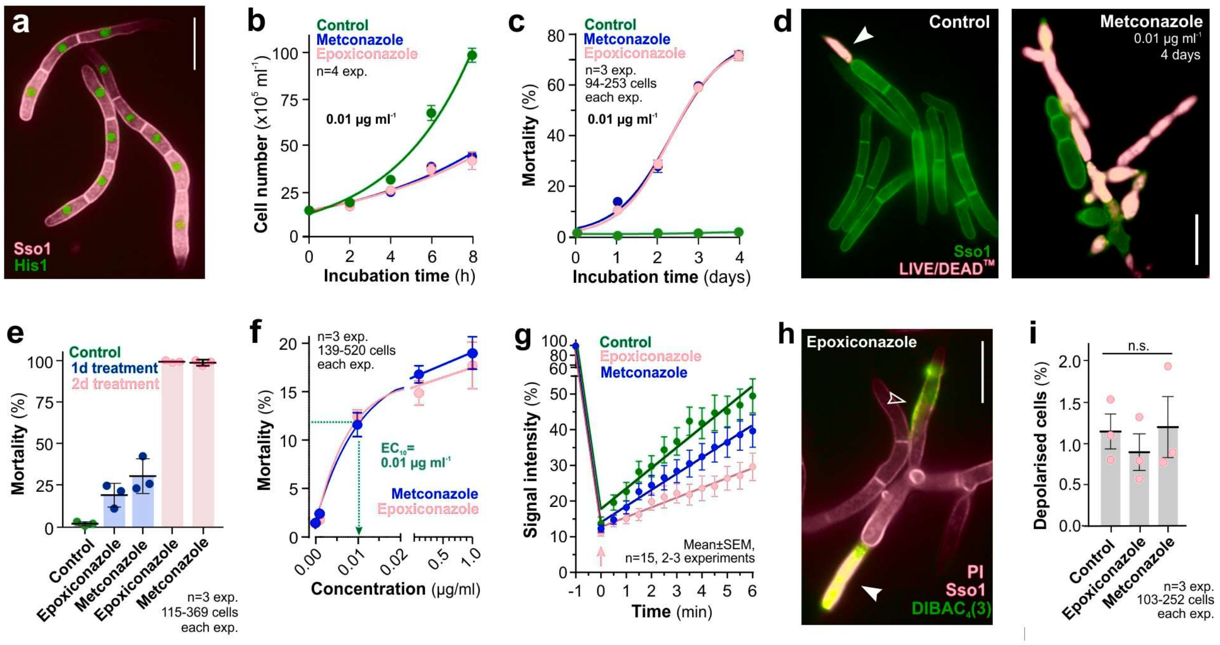

Supplementary Figure 1. Effects of azoles on the plasma membrane in *Z. tritici*.

(**a**) Multi-cellular conidia of *Z. tritici*, expressing the plasma membrane marker mCherry-ZtSso1 (red, Sso1) and the nuclear marker His1-ZtGFP[24] (green, His1). Scale bar= 10 µm.

(**b**) Growth of *Z. tritici* cells in the presence of the solvent (green), 0.01 µg ml^{-1} epoxiconazole (red) and 0.01 µg ml^{-1} metconazole (blue). Note that cells are inhibited in growth, suggesting a fungistatic activity of both azoles. Sample size n=4. See Supplementary Fig. 3a for effect of higher concentrations.

(**c**) Mortality of *Z. tritici* in the presence of the solvent (Control) and 0.01 µg ml^{-1} azoles. Mortality was determined using LIVE/DEAD^{TM} staining. Note that ~70 % of all cells are dead after 4 days of incubation with low concentrations of azoles. Sample size n=3. See Supplementary Fig. 3b, 3c for effect of higher concentrations.

(**d**) LIVE/DEAD^{TM} staining (red) of *Z. tritici* conidia, expressing the plasma membrane marker eGFP-ZtSso1 (green, Sso1), after 4 days incubation with 0.01 µg ml^{-1} epoxiconazole.

(**e**) Mortality after treatment with epoxiconazole or metconazole for 1 day (blue) or 2 days (red), followed by growth for 3 (blue) or 2 days (red) in azole-free medium. Control cells were treated with the solvent methanol. Arrowhead points towards a dead cell. Sample size n= 3.

(**f**) Relative mortality of *Z. tritici* cells after 24 h treatment with various concentrations of epoxiconazole and metconazole. Mortality was assessed using LIVE/DEAD^{TM} staining. The effective concentration at 10 % increase of mortality over background mortality in control sample (effective concentration at 10 %; EC_{10}) is indicated in green. Sample size n= 3.

(**g**) Fluorescent recovery after photo-bleaching eGFP-Sso1 in *Z. tritici* cells, treated with the solvent (Control) and azoles. All linear regression curves fit at R^2 >0.956; both azole recovery slopes are significantly different from control at P<0.0001. Sample size n= 15 cells from 2-3 experiments. See Supplementary Movie 1.

(**h**) Ion permeability of the plasma membrane in epoxiconazole-treated cells. Depolarised cells accumulate $DiBAC_4(3)$ (green; open arrowhead); propidium iodide-positive cells (red, PI) indicated by filled arrowhead; plasma membrane labelled with mCherry-ZtSso1 (red, Sso1)Scale bar= 10µm.

(**i**) Relative number of depolarised cells after 24 h treatment with 0.01 µg ml^{-1} epoxiconazole, metconazole or the solvent methanol (Control). Propidium iodide-positive cells were excluded. Sample size n= 3.

Cells in were grown in YG media at 18°C, 200 rpm, and treated with various concentrations (**b**), or with 0.01 µg ml^{-1} (**c**,**d**,**e**,**g**,**h**,**i**) and 1 µg ml^{-1} (**f**) and grown for 2 h to 4 days; in all control experiments, 0.1 % methanol was used (v v^{-1}). Results shown in (**a,d,h**) were obtained independently in 3 experiments. Values in (**b**,**c**,**d**,**e**,**f**,**g**,**i**) are mean ± standard error of the mean (SEM); dots in (**e**,**i**) represent average of independent experiments; statistical testing in (**i**) used one-way ANOVA testing; n.s.: not significantly different at two-tailed P>0.05; linear and non-linear regression and slope comparison in (**b**,**c**,**d**,**g**) was done in Prism 6.

All data are provided in the Source Data File.

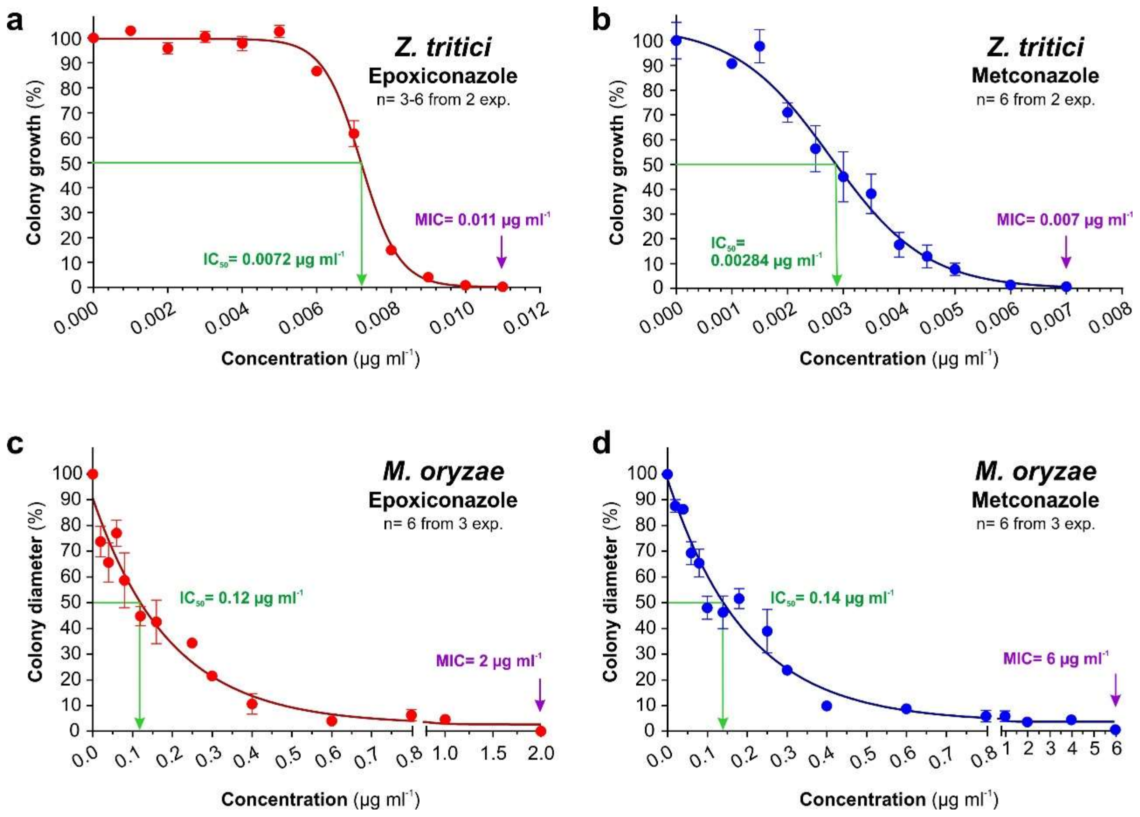

Supplementary Figure 2 Effect of azoles on plate growth of *Z. tritici* and *M. oryzae.*

(**a**,**b**) Growth curves of *Z. tritici*, strain IPO323, grown for 5 days at 18ºC on YPD plates, supplemented with various concentrations of epoxiconazole (**a**) and metconazole (**b**). Concentration at which growth is reduced by 50% (IC_{50}) or completely inhibited (minimal inhibitory concentration = MIC) is indicated. Data for epoxiconazole were taken from Ref. 1.

(**c**,**d**) Curves showing the colony diameter of *M. oryzae*, strain Guy11, grown for 3 days at 25ºC on PDA plates, supplemented with various concentrations of epoxiconazole (**c**) and metconazole (**d**). Concentration at which growth is reduced by 50% (IC_{50}) or completely inhibited (minimal inhibitory concentration = MIC) is indicated.

All data points represent mean ± standard error of the mean; sample size n= 3-6 measurements obtained from 2 (**a**,**b**) and 3 (**c**,**d**) independent experiments; non-linear regression was done in Prism 6.

All data are provided in the Source Data File.

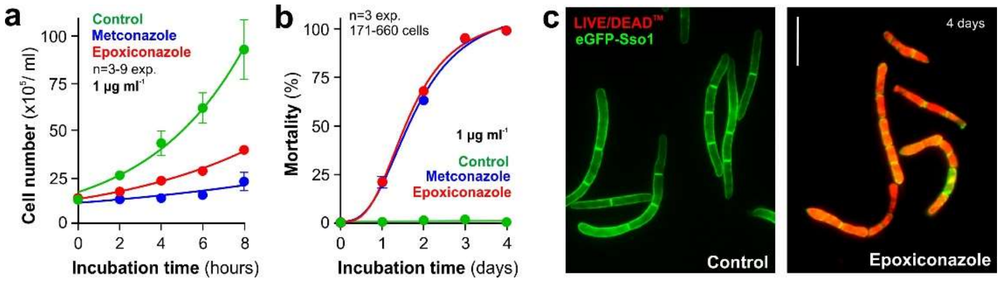

Supplementary Figure 3 Effect of high concentration of azoles on growth and mortality in *Z. tritici*.

(**a**) Growth of *Z. tritici* cells in the presence of the solvent control (green), epoxiconazole (red) and metconazole (blue).

(**b**) Mortality of *Z. tritici* in the presence of the solvent control (green), epoxiconazole (red) and metconazole (blue). Mortality was determined using LIVE/DEAD^{TM} staining.

(**c**) LIVE/DEAD^{TM} staining of *Z. tritici* conidia, expressing the plasma membrane marker eGFP-ZtSso1, after 4 days incubation in the presence of the solvent (Control) or 1 µg ml^{-1} epoxiconazole (Epoxiconazole).

Cells were incubated for 2 - 8 h (**a**) or 1 - 4 days (**b**) with 1 µg ml^{-1} epoxiconazole and metconazole. Results shown in (**c**) were obtained independently in 3 experiments. Data points in (**a**,**b**) are mean ± standard error of the mean; sample size n =3-9 independent experiments (**a**) and n= 3 independent experiments with 171-660 individual cells; non-linear regression was done in Prism 6.

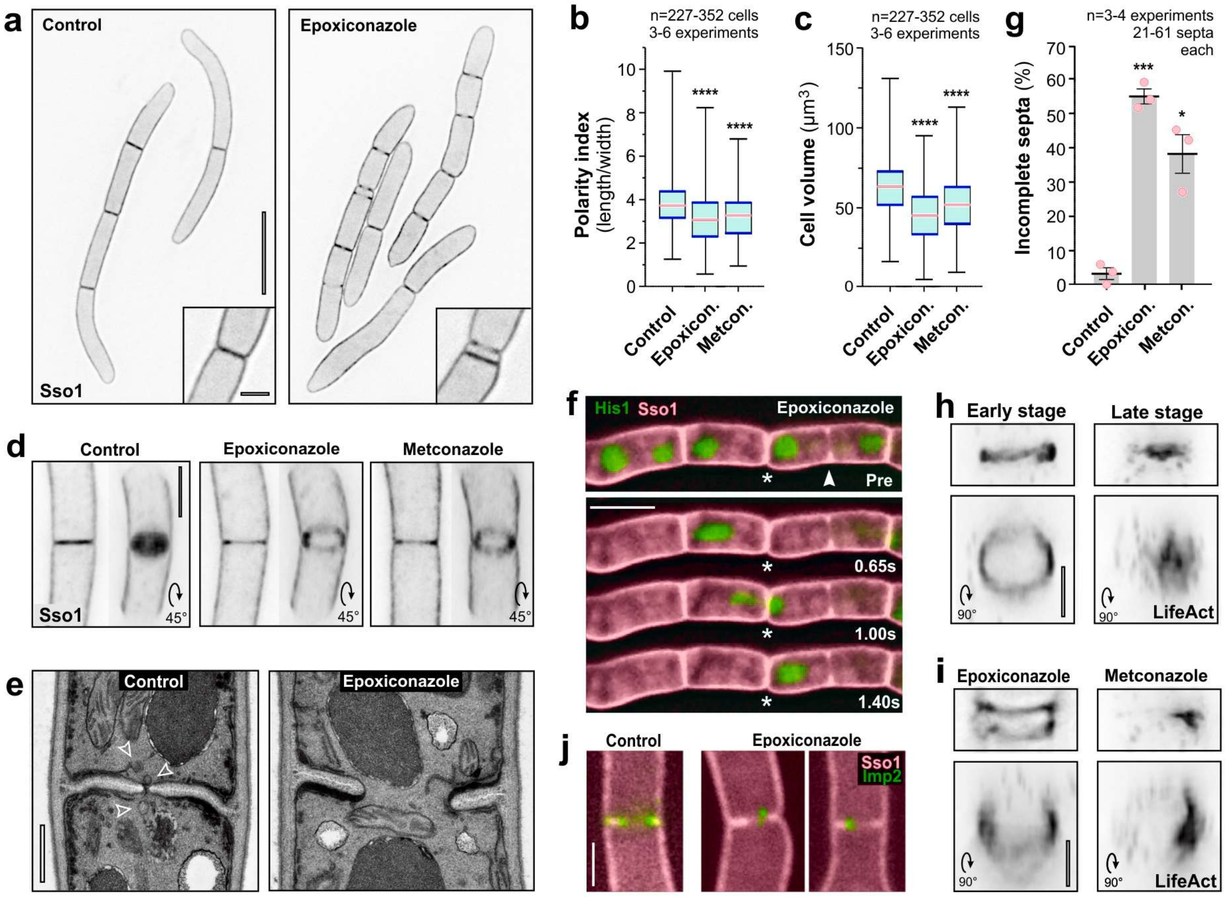

Supplementary Figure 4 Azoles induce incomplete septation.

(**a**) Morphology defect in epoxiconazole-treated *Z. tritici* conidia. The plasma membrane is labelled with eGFP-ZtSso1 (indicated by Sso1); images are contrast inverted. Inserts show mis-positioned septa. Scale bars= 10 µm (overview), 2 µm (inset).

(**b**) Polarity index of control and azole-treated cells. Sample size n=227- 352 cells from 3-6 independent experiments

(**c**) Cell volume in cells treated with solvent (control) or azoles. Calculations assumed a cylindrical shape ($V= \pi r^2 h$). Sample size n=227- 352 cells from 3-6 independent experiments

(**d**) eGFP-ZtSso1-labelled septa in solvent- (Control) and azole-treated cells. Images are contrast inverted. Tilting 3D reconstructions (45°) show open septa. Scale bar= 3 µm. See Supplementary Movies 2.

(**e**) Ultrastructure of septa in solvent- (control) and epoxiconazole-treated cells (epoxiconazole). Woronin bodies are indicated by arrowheads. Scale bar= 0.5 µm.

(**f**) Movement of His1-ZtGFP-labelled nuclei (green; His) through mCherry-ZtSso1-labelled septa (red; Sso1) after laser-wounding of an epoxiconazole-treated conidium. Wounding point is located to the right and not shown. Time after wounding in seconds are indicated; "Pre": before laser treatment; asterisk shows septum that appears intact; arrowhead shows incomplete septum. Scale bar= 5 µm. See Supplementary Movie 3.

(**g**) Number of “open” septa identified by passage of nuclei after laser wounding in solvent (Control) and azole-treated conidia. Sample size n= 3- 4 independent experiments with 21-61 septa per experiment.

(**h**, **i**) F-actin rings, labelled with LifeAct-ZtGFP (indicated by LifeAct) in untreated (**h**) and azole-treated (**i**) conidia. Images are contrast inverted; 90°-tilted images provided. Scale bar= 2µm. See Supplementary Movie 5.

(**j**) Localisation of the putative F-BAR protein ZtImp2-ZtGFP (green, Imp2) at septa in methanol-treated cells (Control) and epoxiconazole-treated cells (Epoxiconazole). The plasma membrane is labelled with mCherry-Sso1 (red; Sso1). Scale bar= 2 µm. See Supplementary Fig. 5 and Supplementary Movie 4.

Cells were grown in YG media at 18°C, 200 rpm, and treated with 0.01 µg ml^{-1} of the azoles for 24 h. Results shown in (**a,d,f**) were obtained independently in 3 experiments and (**e,h,I,j**) in 2 experiments. Data in (**b**,**c**) did not pass a normality test (Shapiro-Wilk test, all P-values <0.0035) and are given as Whiskers' plots with 25/75 percentiles (blue lines), median (red line) and minimum and maximum (whiskers ends); bars in (**g**) represent mean ± SEM; red dots represent independent experiments; statistical analysis in (**b**,**c**) was done using non-parametric Mann-Whitney testing, in (**g**) Student's t-testing; *= different from control at two-tailed P= 0.0203 (**g**); ***= two-tailed P= 0.0001 (**g**); ****= two-tailed P< 0.0001 (**b**,**c**).

All data are provided in the Source Data File.

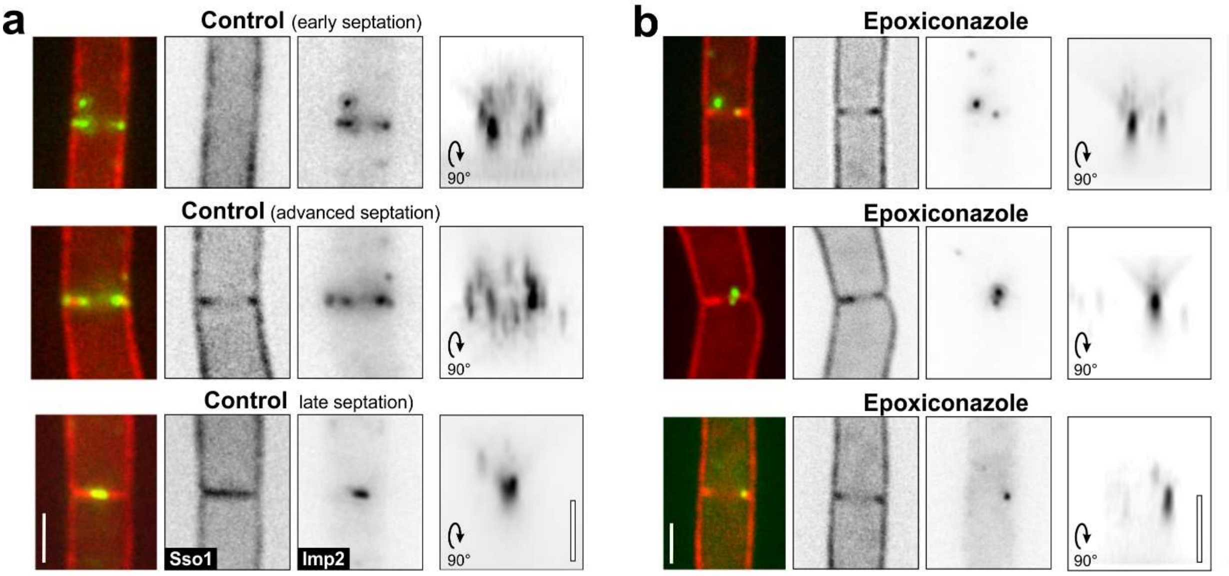

Supplementary Figure 5 Localisation of an Imp2 homologue in epoxiconazole-treated *Z. tritici* cells.

(**a**) A fusion protein of ZtGFP and ZtImp2 (Imp2), a close *Z. tritici* homologue of *S. pombe* Imp2, localises to the growing septum, visualised by mCherry-ZtSso1 (Sso1). The ring appears before the septum is formed and constricts in late septation. This localisation is consistent with a role of the putative F-BAR protein in anchoring the contractile actin ring to the plasma membrane[2]. All black-white images are contrast inverted. 90°-tilted images were obtained from 3D reconstructions of Z-axis image stacks. Scale bars= 2 µm.

(**b**) Localisation of a fusion protein of ZtGFP and the putative F-BAR protein ZtImp2 (Imp2) at incomplete septae in epoxiconazole-treated *Z. tritici* spores. Few ZtGFP-ZtImp2 signals are located at the region of septum formation. All black-white images are contrast inverted. 90°-tilted images were obtained from 3D reconstructions of Z-axis image stacks. Scale bars= 2 µm. Results were obtained independently in 2-3 experiments.

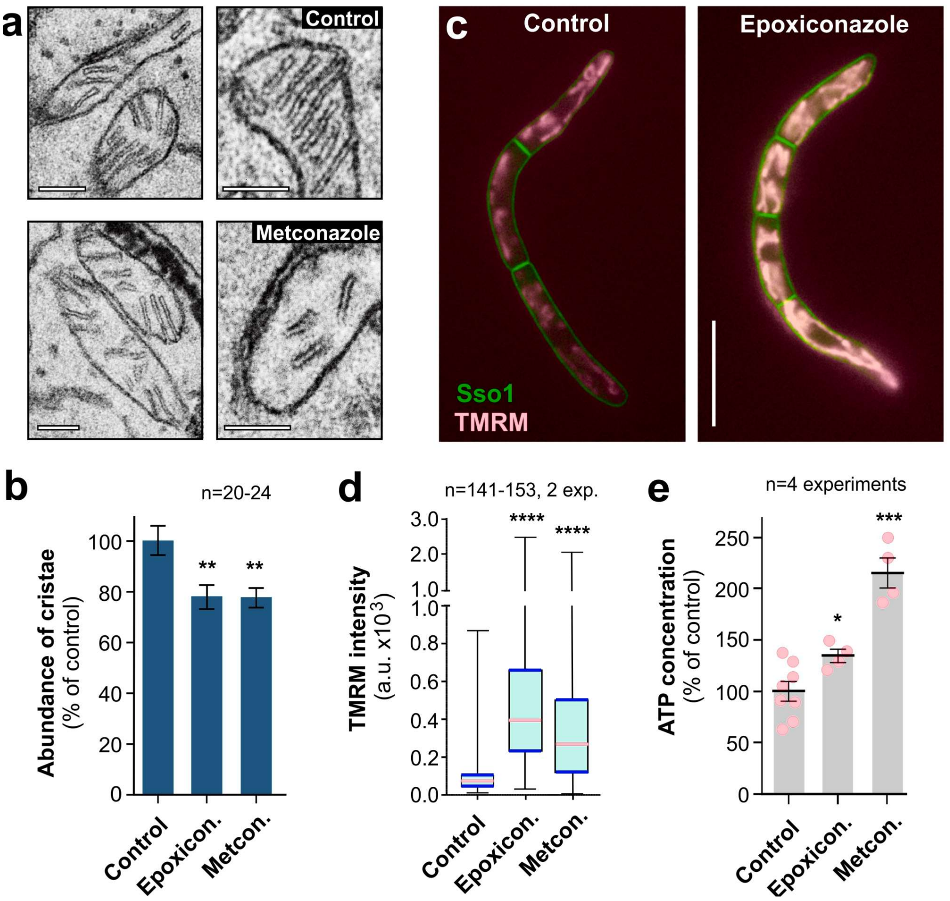

Supplementary Figure 6. Azoles hyperpolarise mitochondria and increase cellular ATP.

(**a**) Ultrastructure of *Z. tritici* mitochondria, treated with 0.01 µg ml^{-1} metconazole for 24 h. Scale bars= 0.2 µm (left images) and 0.15 µm (right images). See Supplementary Fig. 7a for mitchondria in control cells.

(**b**) Relative abundance of cristae in control and azole-treated mitochondria. Data derived from electron microscopy images. Area of cristaer per area in control cell was set to 100 %. Sample size n= 20-24 from 2 independent experiments

(**c**) Mitochondrial membrane potential (red, TMRM) in eGFP-ZtSso1-expressing cells (green, Sso1). Images were acquired using the same microscopic settings and were image processed identically. Scale bar= 10 µm. See Supplementary Fig. 7b.

(**d**) Mitochondrial membrane potential, determined by quatitative TMRM staining, in solvent- (Control), epoxioconazole- (Epoxicon.) or metconazole- (Metcon.) treated cells. Cells were incubated for 24 h at 0.01 µg ml^{-1} azoles or the solvent. Sample size of 141-153 cells from 2 independent experiments.

(**e**) Cytplasmic ATP concentration in *Z. tritici* cells, treated with the solvent methanol (Control), epoxiconazole (Epoxicon.) and metconazole (Metcon.). Sample size n= 4 independent experiments.

All experiments were done using cells that were treated for 24 h with 0.01 µg ml^{-1} azoles. Results shown in (**a, c**) were obtained independently in 2 experiments. Data in (**d**) did not pass a normality test (Shapiro-Wilk test, all P-values <0.0001) and are given as Whiskers' plots with 25/75 percentiles (blue lines), median (red line) and minimum and maximum (whiskers ends); bars in (**b**,**e**) represent mean ± SEM; and (**e**), with red dots representing values from independent experiments; statistical analysis in (**d**) was done using non-parametric Mann-Whitney testing, and in (**b**,**e**) Student's t-testing after Welch's correction; *= significant difference to control at two-tailed P= 0.0114 (**e**); **= significant difference to control at two-tailed P= 0.0057 (**b**, left bar) and P= 0.0026 (**b**, right bar); ***= significant difference to control at two-tailed P= 0.0008 (**e**); ****= significant difference to control at two-tailed P<0.0001 (**d**). All data are provided in the Source Data File.

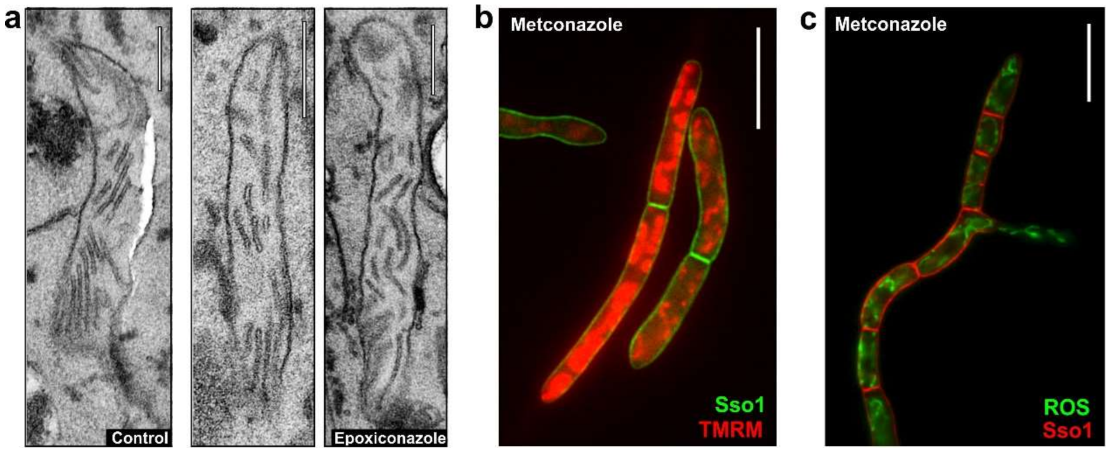

Supplementary Figure 7 Mitochondria organization, hyper-polarization and ROS development in azole-treated *Z. tritici* cells.

(**a**) Ultrastructure of cell treated with 0.1% v v^{-1} methanol (Control) and 0.01 µg ml^{-1} epoxiconazole for 24 h. Scale bars= 0.3 µm.

(**b**) Staining of mitochondrial membrane potential in cells treated with 0.01 µg ml^{-1} metconazole for 24 h, using the dye TMRM (red); the plasma membrane is labelled by eGFP-ZtSso1 (green). Scale bar= 10 µm.

(**c**) Staining of mitochondrial ROS in cells treated with 0.01 µg ml^{-1} metconazole, for 24 h using the dye DHR-123 (green); the plasma membrane is labelled by mCherry-ZtSso1 (red). Scale bar= 10 µm.

All results shown were obtained independently in 2 experiments. All data are provided in the Source Data File.

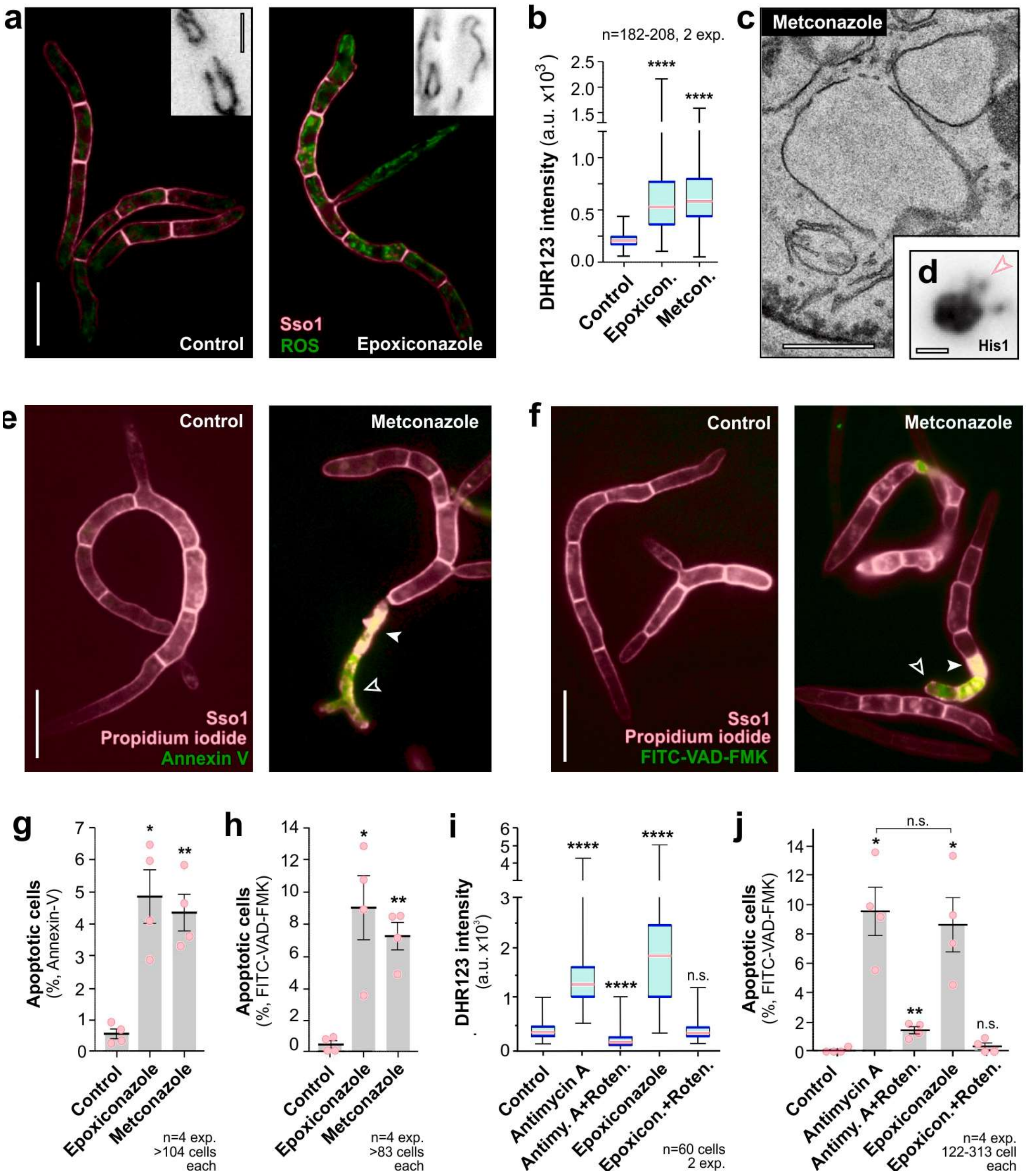

Supplementary Figure 8. Azoles induce mROS-dependent apoptosis.

(**a**) DHR123 staining of mROS (green) in control and epoxiconazole-treated cells. The plasma membrane is labelled withy mCherry-ZtSso1 (red, Sso1); insert shows contrast-inverted images of DHR123-stained mitochondria at higher magnification. Images were acquired using the same microscopic settings and were image processed identically. Scale bars= 10 µm and 3 µm (inset). See also Supplementary Fig. 7c.

(**b**) mROS levels, given as DHR123 fluorescence, in solvent- (Control) and azole-treated cells (Epoxicon., Metcon.). Sample size n= 182-208 cells from 2 independent experiments.

(**c**) Electron micrograph of nuclear fragments in metconazole-treated cells. Scale bars= 0.5 µm. See Supplementary Fig. 9a, 9b.

(**d**) Contrast-inverted image of a nucleus, labelled with DNA-binding His1-ZtGFP, in a metconazole-treated cell. Arowhead indicates "fuzzy" DNA fragments. Scale bar= 1 µm.

(**e**,**f**) Staining of apoptotic cells with Annexin-V (**d**, green) and FITC-VAD-FMK (**e**, green) in control and metconazole-treated cells. Propidium iodide-positive dead cells indicated by filled arrowhead (red, PI). The plasma membrane is labelled with mCherry-ZtSso1 (red, Sso1). Scale bars= 10µm. See also Supplementary Fig. 9c, 5d.

(**g**,**h**) Proportion of apoptotic cells, visualised with Annexin-V (**g**) and FITC-VAD-FMK (**h**) in control and azole-treated cells. Sample size n= 4.

(**i**) mROS levels in cells treated with epoxiconazole and inhibitors of mitochondrial respiration complex I (rotenone= Roten.) and complex III (Antimycin A= Antimy. A). Sample size n= 60 from 2 independent experiments.

(**j**) Number of apoptotic cells after treatment with mROS-inducing Antimycin A (Antimycin A), Antimycin A + rotenone (Antimy.+Roten.), epoxiconazole (Epoxiconazole) and rotenone (Epoxicon.+Roten.). Apoptotic cells were identified by FITC-VAD-FMK staining. Sample size n= 4.

Cells were grown in YG media at 18°C and treated with 0.01 µg ml^{-1} azoles for 24 h; for (**i,j**), cells were additional treated with 100 µM rotenone or 10 µM antimycin A for 24 h. Results shown in (**a,c,d**) were obtained independently in 2 experiments and

(**e**,**f**) from independently in 4 experiments. Data in (**b**,**i**) did not pass a normality test (Shapiro-Wilk test, all P-values <0.0128) and are given as Whiskers' plots with 25/75 percentiles (blue lines), median (red line) and minimum and maximum (whiskers ends); bars in (**g,h**,**j**) represent mean±SEM; with red dots representing values from independent experiments; statistical analysis in (**b**,**i**) was done using non-parametric Mann-Whitney testing, and in (**g**,**h**,**j**) Student's t-testing; n.s.= non-significant difference to control at two-tailed P>0.05; *= two-tailed P= 0.0127 (**g**), P= 0.0227 (**h**), P= 0.0188 (**j**, Control vs. epoxiconazole) and P= 0.0103 (**j**, Control vs. antimycin A); **= two-tailed P= 0.0052 (**g**), P= 0.0029 (**h**) and P= 0.0083 (**j**); ****= two-tailed P<0.0001 (**b**,**i**).

All data are provided in the Source Data File.

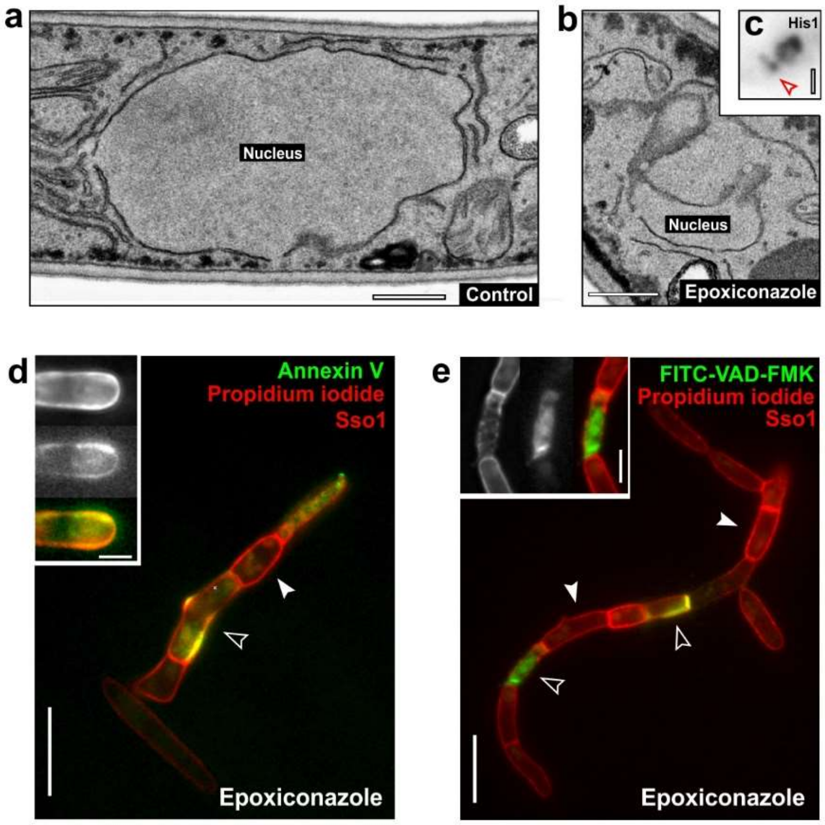

Supplementary Figure 9 Indications of apoptosis in epoxiconazole-treated *Z. tritici* cells.

(**a**) Ultrastructure of a nucleus in a control cell. Scale bar= 0.5 µm.

(**b**) Nuclear fragments in epoxiconzole-treated cells. Scale bars= 0.5 µm.

(**c**) Contrast-inverted image of His1-ZtGFP in a disintegrating nucleus (fragments indicated by red arrowhead). Scale bars= 1 µm.

(**d**) Visualising apoptotic *Z. tritici* cells by annexin-V-fluorescein staining. Healthy cells are unstained (closed arrowhead; only red-fluorescent plasma membrane marker mCherry-ZtSso1 visible), whereas apoptotic cells expose phosphatidylserine and show green-fluorescent at the plasma membrane (overlay with mCherry-ZtSso1 results in yellow colour; open arrowhead). Dead post-apoptotic cells were identified by double staining with propidium iodide, which resulted in red-fluorescent cytoplasmic staining of dead cells (not shown). These dead cells were excluded from the analysis. Inset shows second example of annexin-V-stained cell. Scale bars = 10 µm (overview) and 3 µm (inset).

(**e**) Visualising apoptotic *Z. tritici* cells by FITC-VAD-FMK staining. Healthy cells exclude the dye (closed arrowheads). Azoles induce programmed cell death (24 h treatment), with apoptotic cells being filled with green-fluorescence (open arrowheads). Cells were double-stained with propidium iodide to identify post-apoptotic dead cells, which were not included in the analysis. The plasma membrane is visualised by the marker mCherry-ZtSso1. Inset shows higher magnification and individual grey-scale images, used for the overlay image. Scale bars= 10 µm (overview) and 3 µm (inset).

Results shown in (**a**-**c**) were obtained independently in 2 experiments and (**d**,**e**) were obtained independently in 4 experiments. All data are provided in the Source Data File.

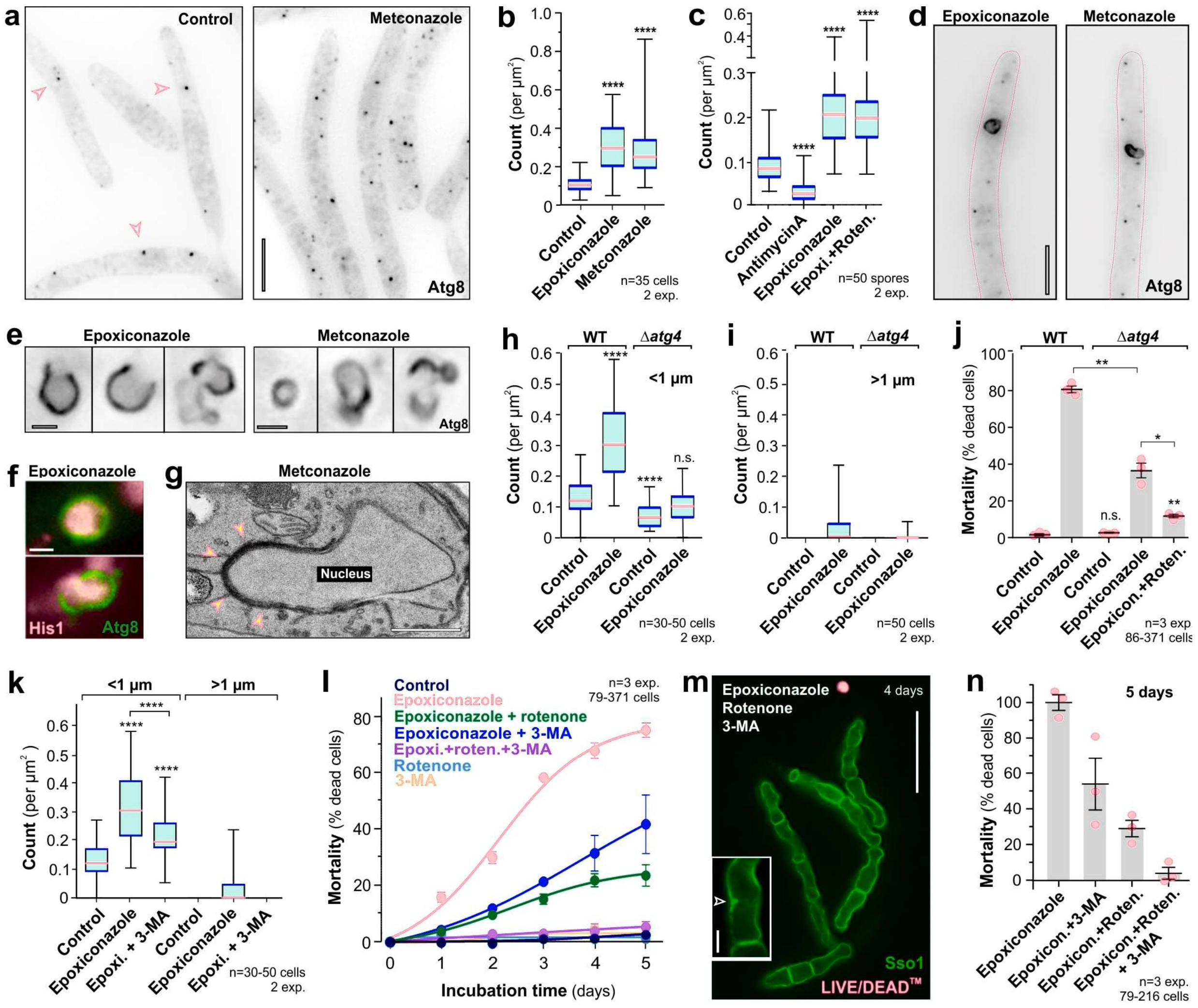

Supplementary Figure 10. Azoles kill *Z. tritici* cells by inducing apoptosis and autophagy.

(**a**) Contrast inverted maximum-projection of autophagosomes, labelled with eGFP-Atg8 (Atg8) in cells treated with solvent (Control) or metconazole; arrowheads indicate individual autophagosomes. Scale bar= 5 µm.

(**b**) Number of autophagosomes in control and azole-treated cells. Sample size n= 35 cells from 2 independent experiments.

(**c**) Number of autophagosomes under increased mROS levels (AntimycinA) and epoxiconazole-treated cells with reduced mROS levels. mROS is not controlling autophagosome numbers. Sample size n= 50 cells from 2 independent experiments.

(**d,e**) Contrast-inverted maximum projections of large autophagosomes, labelled with eGFP-Atg8 in azole-treated cells. The cell border in (**d**) is indicated by red dotted line. Scale bar= 5 µm (**d**) and 1 µm (**e**). See Supplementary Movie 6.

(**f**) Co-visualisation of eGFP-Atg8 (green, Atg8) and His1-mCherry (red, his1) in living epoxiconazole-treated cells. Scale bar= 1 µm. See Supplementary Fig. 11b.

(**g**) Ultrastructure of a putative large autophagosome (arrowheads), engulfing a nucleus in a metconazole-treated cell. Scale bar= 0.5 µm. See also Supplementary Fig. 11d.

(**h**,**i**) Small autophagosomes (<1 µm; **h**) and large autophagosomes (>1 µm; **i**) in wildtype (WT) and Δ*atg4* mutants, treated with solvent (control) and azoles. Sample size n= 30-50 cells from 2 independent experiments.

(**j**) Mortality of epoxiconazole-treated *Z. tritici* cells in wildtype and Δ*atg4* mutant background. Sample size n= 3 independent experiments with 86-371 cells.

(**k**) Effect of the autophagy inhibitor 3-MA on epoxiconazole-induced autophagosome formation. Sample size n= 30-50 cells from 2 independent experiments.

(**l**) Mortality of *Z. tritici* cells, treated for 1-5 days with the solvent (Control), epoxiconazole, rotenone or 3-MA and various combinations of these compounds. Sample size n= 3 independent experiments with 79-371 cells.

(**m**) eGFP-Sso1-expressing cells (green, Sso1), co-treated with epoxiconazole, 3-MA and rotenone (4 days) and stained with LIVE/DEAD^{TM} dye (red). Inset shows an incomplete septum in the inhibitor-treated cells. Scale bars= 10 µm (overview), 3 µm (inset).

(**n**) Mortality after 5 days treatment with epoxiconazole alone, or in combination with the inhibitors 3-MA and rotenone. Data taken from (**l**) and corrected for background mortality in cultures grown for 5 days in the presence of the solvent (control). Sample size n= 3 independent experiments with 79-216 cells.

Cells were grown in YG media, 18°C, 200 rpm and treated 24 h with 0.01 µg ml^{-1} azoles or as indicated; rotenone was used at 100 µM and 3-MA at 5 µM for 24 h. Results shown in (**a,d,e,f,g**) were obtained independently in 2 experiments and (**m**) from 3 experiments. Data in (**j,l,n**) given as mean ± SEM, with red dots representing

values from independent experiments; some data sets in (**b**,**c**,**h**,**i**,**k**) did not pass a normality test (Shapiro-Wilk test; P<0.05) and thus all data are given as Whiskers' plots with 25/75 percentiles (blue lines), median (red line) and minimum and maximum (whiskers ends); statistical analysis in (**b**,**c**,**h**,**i**,**k**) used non-parametric Mann-Whitney testing; (**j**) used Student's t-testing with Welch correction; data in symbols above whiskers indicate comparison with control; brackets indicate other comparisons; n.s.= non-significant difference two-tailed P>0.05; *= two-tailed P= 0.0202 (**j**); **= two-tailed P= 0.0026 (**j**, WT "Epoxiconazole" compared to Δ*atg4* "Epoxiconazole") and P= 0.0069 (**j**, Δ*atg4* "Epoxicon.+Roten." versus Δ*atg4* "Control"); ****= two-tailed P<0.0001 (**b,c**,**h**,**k**).

All data are provided in the Source Data File.

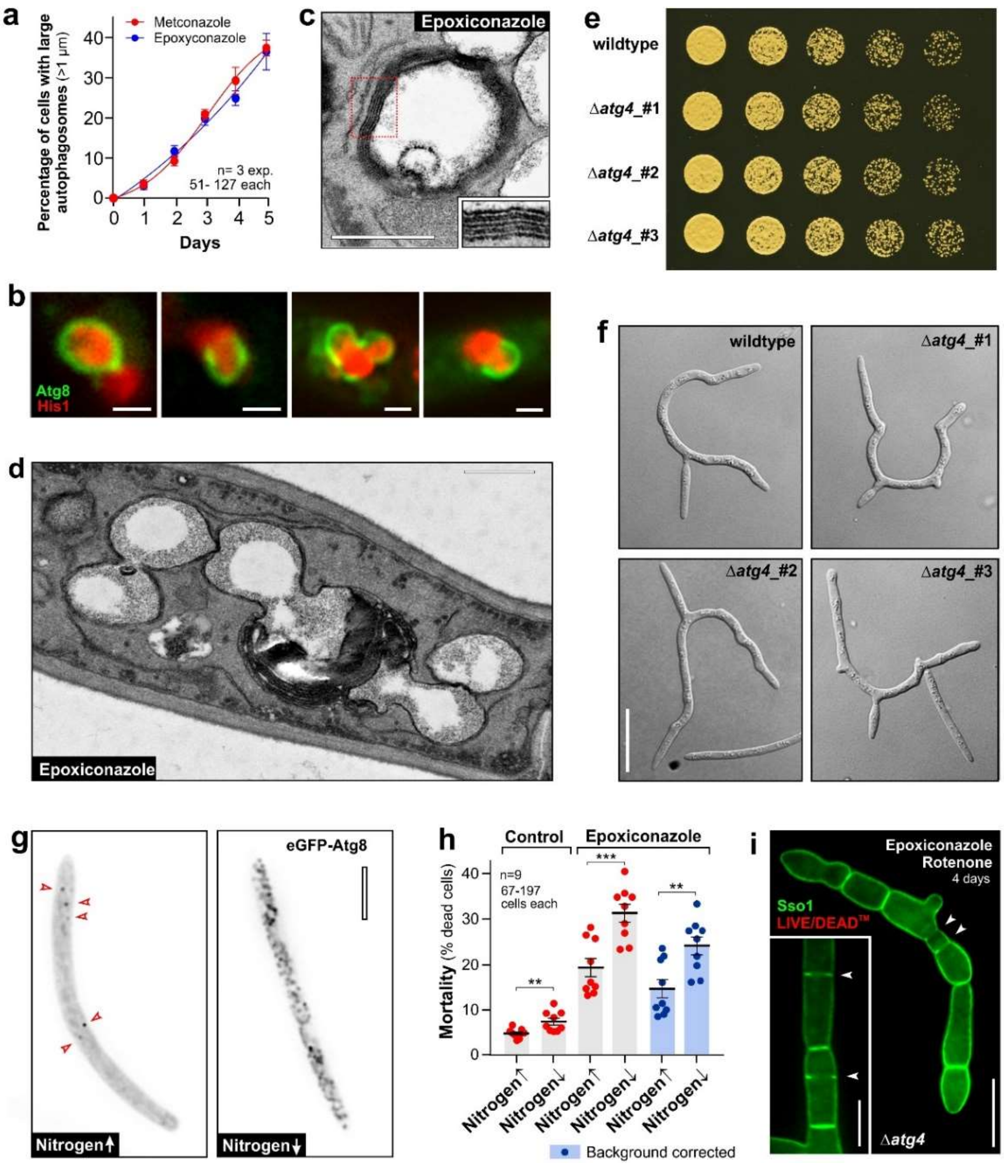

Supplementary Figure 11 Data supporting results around azole-induced autophagy in *Z. tritici* cells.

(**a**) Development of large autophagosomes. eGFP-Atg8-positive structures of >1 µm diameter) with time in the presence of 0.01 µg ml^{-1} epoxiconazole or metconazole. Non-linear regression was done in Prism 6.

(**b**) Examples of large marcoautophagosomes, visualised with eGFP-Atg8 and His1-mCherry in living cells of *Z. tritici*, treated for 24 h with 0.01 µg ml^{-1} epoxiconazole. Scale bar= 1 µm.

(**c**) Autophagosome in an epoxicoazole-treated *Z. tritici* cell. Insert shows boxed region in overview. Inset shows higher magnification of the membranous coating of

the organelle. The organelle is surrounded by multiple membranes. Scale bar= 0.5 µm.

(**d**) Ultrastructure of autophagosomes. Scale bar= 0.5 µm.

(**e**) Plate growth of *Z. tritici* strain IPO323 (wildtype) and 3 independent *atg4* deletion mutants (Δ*atg4*_#1-3). The autophagy-impaired mutants show no obvious growth defect.

(**f**) Morphology of conidia in *Z. tritici* strain IPO323 (wildtype) and 3 independent *atg4* null mutants (Δ*atg4*_#1-3). The autophagy-impaired mutants show no aberrant morphology. Scale bar= 20 µm.

(**g**) Example images, showing eGFP_Atg8-positive autophagosomes in *Z. tritici* cells grown in minimal medium, supplemented with $NaNO_3$ as nitrogen source (Nitrogen↑) and in minimal medium without nitrogen (Nitrogen↓). Scale bar= 5 µm.

(**h**) Cell mortality in *Z. tritici* conidia, grown in minimal medium, supplemented with $NaNO_3$ as nitrogen source (Nitrogen↑) and in minimal medium without nitrogen (Nitrogen↓) and treated with solvent (Control) or 0.01 µg ml^{-1} epoxiconazole for 24 h. Mortality was determined using LIVE/DEAD™ staining

(**i**) eGFP-Sso1-expressing Δ*atg4* mutant conidium (green), treated with epoxiconazole and rotenone and stained with LIVE/DEAD™ dye (red). Arrowheads indicate incomplete septum. Scale bar= 5 µm (inset) and 10 µm (overview).

Cells in (**g**,**h**) were grown in minimal medium with or without $NaNO_3$. Results shown in (**b,c**,**d**,**f,g**) were obtained independently in 2 experiments and (**e,i**) were obtained independently in 3 experiments. Data in (**a**,**h**) are given as mean ± standard error of the mean with a sample size n=3 with 51-127 cells each (**a**) and n= 9 with 67-197 cells each, individual experiments are indicated by dots (**h**); statistical testing used Student's t-test with Welsh correction; **= significant difference between data sets at two-tailed P-value 0.0088 (**h**, Control (Nitrogen↑) vs Control (Nitrogen↓) and 0.0041 [**h**, Epoxiconazole (Nitrogen↑) vs Epoxiconazole (Nitrogen↓) background corrected]; ***= significant difference between data sets at two-tailed P-value 0.0006 (**h**).

All data are provided in the Source Data File.

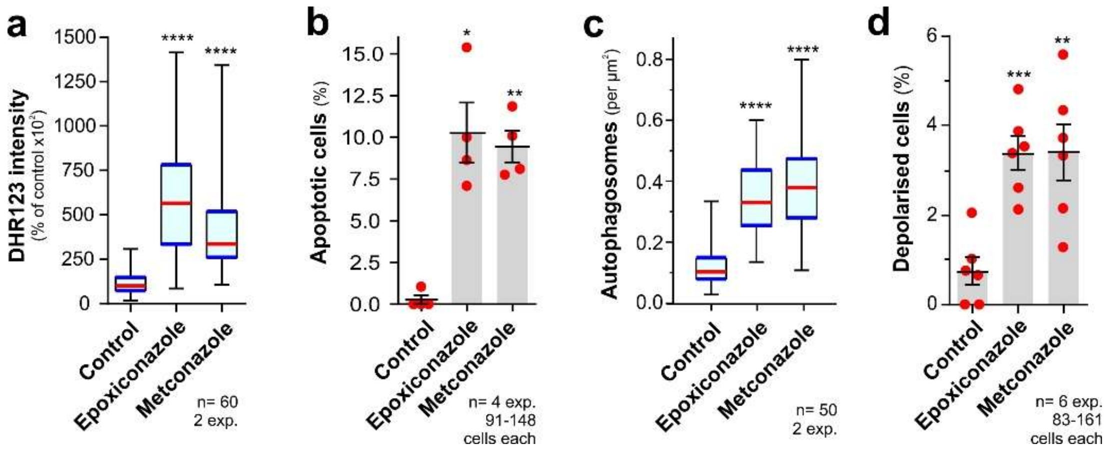

Supplementary Figure 12 MoA of azoles at higher concentrations in *Z. tritici*.

(**a**) mROS, given as DHR-123 fluorescence, in cells treated with 1.0 µg ml^{-1} azoles or the solvent methanol (Control).

(**b**) Induction of apoptosis, shown as relative number of FITC-VAD-FMK-stained cells after 24 h, in cells treated with 1.0 µg ml^{-1} azoles or the solvent methanol (Control).

(**c**) Induction of autophagy, shown as the number eGFP-Atg8-labelled autophagosomes in 1 $µm^2$ of a maximum projection of a focal plane in cells treated with 1.0 µg ml^{-1} azoles or the solvent methanol (Control).

(**d**) Relative number of depolarised cells after 24 h treatment with 1.0 µg ml^{-1} azoles or the solvent methanol (Control). Propidium iodide-positive cells were excluded.

Cells were treated for 24 h with 1 µg ml^{-1} epoxiconazole or metconazole. Some data sets in (**a**) and (**c**) did not pass a normality test (Shapiro-Wilk test; P<0.05) and thus all data are given as Whiskers' plots (blue lines: 25/75 percentiles; red line: median) with an sample size n= 60 (**a**) and 50 (**b**)) from 2 independent experiments; statistical analysis in (**a**) and (**c**) used non-parametric Mann-Whitney testing; **** indicates statistical difference to control at two-tailed P value <0.0001; data in (**b**,**d**) are given as mean ± standard error of the mean with an sample size n= 4 (**b**) and 6 (**d**), individual experiments are indicated by red dots; statistical testing in (**b**,**d**) used Student's t-test after Welch's correction; *= significant difference to control at two-tailed P= 0.0106 (**b**); **= significant difference to control at two-tailed P= 0.0014 (**b**) and 0.0063 (**d**); ***= significant difference to control at two-tailed P= 0.0004 (**d**); ****= significant difference to control at two-tailed P<0.0001 (**d**).

All data are provided in the Source Data File.

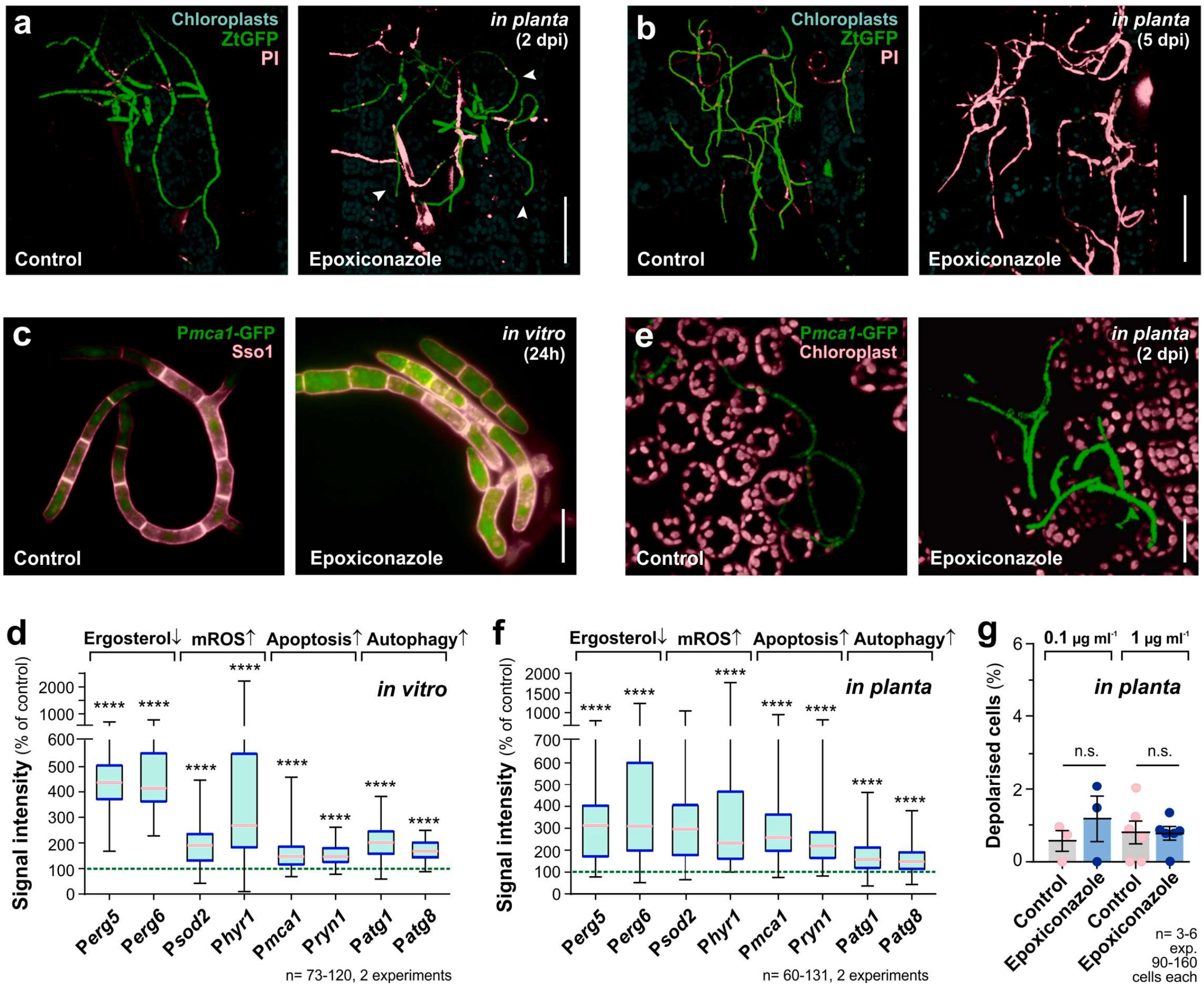

Supplementary Figure 13. Activity of epoxiconazole in *Z. tritici* during early plant infection.

(**a**, **b**) Epoxiconazole-induced mortality of *Z. tritici*, expressing cytoplasmic codo-optimised GFP (green, ZtGFP) on the plant leaf surface. Wheat leaves were pre-treated with the solvent methanol (Control) or 0.1 µg ml^{-1} epoxiconazole, followed by inoculation with pathogen conidia 24 h later. LIVE/DEAD staining using propidium iodide was performed 2 days (**a**) and 5 days (**b**) later. Dead cells appear red, green cells are alive. Arrowheads indicate hyphae. Scale bars= 50 µm.

(**c**) Expression of ZtGFP under the control the promoter of the metacaspase gene *mca1* (green, P*mca1*-ZtGFP) in liquid culture; the PM is labelled with mCherry-ZtSso1 (red, Sso1). Expression of *mca1* is induced in the presence of epoxiconazole, resulting in green-fluorescent cytoplasm. Images were acquired

using the same microscopic settings and were image processed identically. Scale bars= 10 µm.

(**d**) Quantitative analysis of cytoplasmic green fluorescence in liquid culture-grown marker strains exposed to epoxiconazole. The marker strains respond to reduced ergosterol levels (P*erg5*, P*erg6*), increased mROS (P*sod2*, P*hyr1*), apoptosis (P*mca1*, P*ryn1*) or autophagy (P*atg1*, P*atg8*) by inducing ZtGFP expression. The green-dotted line represents the median of fluorescent intensity in cells exposed to the solvent alone; this value was set to 100 %. Sample size n= 73-120 cells from 2 independent experiments. For further information on marker genes see Supplementary Table 4.

(**e**) Expression of ZtGFP under the control the promoter of the metacaspase gene *mca1* (green, P*mca1*-ZtGFP); chloroplast autofluorescence is shown in red. Expression of *mca1* is induced in the presence of epoxiconazole, resulting in green-fluorescent cytoplasm. Images were acquired using the same microscopic settings and were image processed identically. Scale bars= 20 µm.

(**f**) Quantitative analysis of cytoplasmic green-fluorescence in marker strains that rested on leaf surfaces for 2 days. The leaves were pre-sprayed with epoxiconazole or the solvent methanol (control). The green-dotted line represents the median of fluorescent intensity in control cells; this value was set to 100 %. Sample size n= 60-131 cells from 2 independent experiments. For further information on marker genes see Supplementary Table 4.

(**g**) Relative number of depolarised *Z. tritici* cells on plant surfaces. Propidium iodide-positive cells (=dead cells) were excluded from the analysis. Sample size n= 3-6 independent experiments with 60-131 cells each.

For *in vitro* experiments (**c**,**d**), cells were treated for 24 h with 0.01 µg ml^{-1} epoxiconazole; for *in planta* experiments, 12 day-old wheat plants were sprayed with 0.1 µg ml^{-1} (**a**,**b**,**e**,**f**,**i**,**g**) or 1 µg ml^{-1} (**g**) epoxiconazole and imaged at 1 dpi (**g**), 2 dpi (**a**,**e**,**f**) or 5 dpi (**b**). Results shown in (**a**,**b**,**c**,**e**) were obtained independently in 2 experiments. . Data in (**g**) given as mean ± SEM, with dots representing values from independent experiments, some data sets in (**d**, **f**) did not pass a normality test (Shapiro-Wilk test; P<0.05) and thus all data are given as Whiskers' plots with 25/75 percentiles (blue lines), median (red line) and minimum and maximum (whiskers

ends). Statistical analysis in (**e**, **f**) used non-parametric Mann-Whitney testing and in (**g**) Student's t-testing; n.s.= not significantly different at two-tailed P>0.05; ****= statistical difference to control at two-tailed P value <0.0001.

All data are provided in the Source Data File.

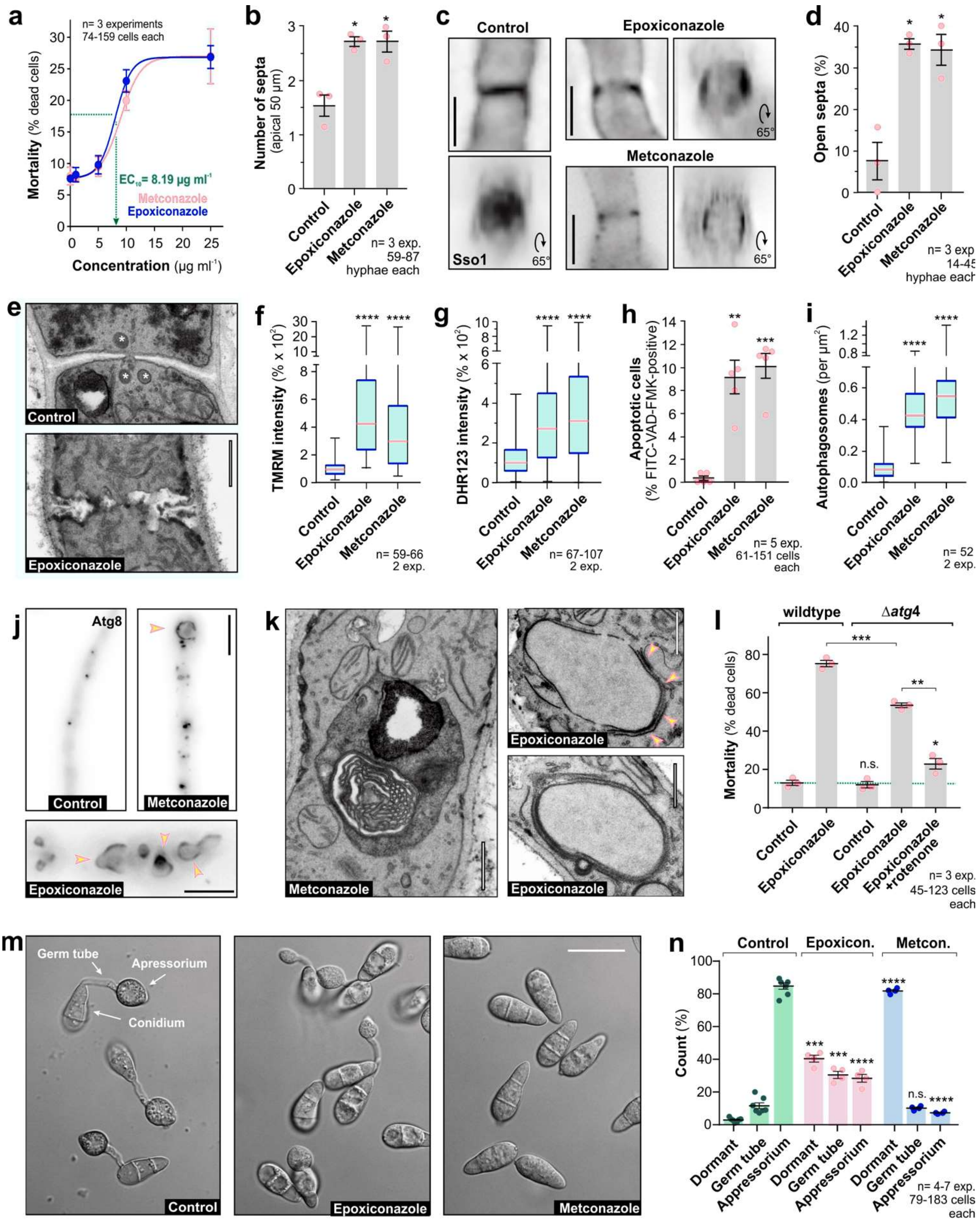

Supplementary Figure 14. The rice blast pathogen *M. oryzae* shares the azole MoA with *Z. tritici*.

(**a**) Mortality of hyphal *M. oryzae* cells, treated with azoles for 24 h. The effective concentration at 10 % increase of mortality over background mortality in control sample (EC_{10}) is indicated in green. Sample size n= 3 independent experiments with

74-159 cells each. See also Supplementary Fig. 15 for mortality at higher concentrations and Supplementary Fig. 2c, 2d for growth inhibition on agar plates.

(**b**) Number of septa, labelled with *M. oryzae* Sso1, fused to eGFP (Sso1-eGFP) in the hyphal apical 50 µm, treated with solvent (Control) and azoles. Sample size n= 3 independent experiments with 59-87 hyphae each.

(**c**) Sso1-eGFP-labelled septa (Sso1) in solvent- (control) and azole-treated hyphae. Images are contrast inverted. Tilting 3D reconstructions are given (65°). Scale bars= 3 µm. See Supplementary Movie 7.

(**d**) Incomplete septae in hyphae, treated with solvent (Control) and azoles. Sample size n= 3 independent experiments with 14-45 hyphae each.

(**e**) Ultrastructure of septa in solvent- (control) and epoxiconazole-treated hyphae. Woronin bodies indicated by asterisks. Scale bar= 0.5 µm. See also Supplementary Fig. 16.

(**f**) IMM potential, given as TMRM fluorescence, in hyphae treated with solvent (Control) and azoles. Sample size n= 59-66 cells from 2 independent experiments.

(**g**) mROS levels, given as DHR-123 fluorescence, in hyphae treated with solvent (Control) and azoles. Sample size n= 67-107 cells from 2 independent experiments.

(**h**) Apoptosis, shown as relative number of FITC-VAD-FMK-stained hyphae after 24 h treatment with solvent (Control) and azoles. Sample size n= 5 independent experiments with 61-1581 cells each

(**i**) eGFP-Atg8-labelled autophagosomes in hyphae*,* treated with solvent (Control) and azoles. Sample size n= 52 cells from 2 independent experiments.

(**j**) Autophagosomes, labelled with green-fluorescent *M. oryzae* Atg8 (Atg8) in hyphae that were treated with solvent (Control) and azoles. Arrowheads indicate large autophagosomes. Scale bars= 5 µm (upper panels) and 3 µm (lower panel). Images contrast-inverted.

(**k**) Ultrastructure of autophagosomes in azole-treated hyphae. Arrowheads indicate phagosome surrounding a nucleus. Scale bar= 0.5 µm.

(**l**) Mortality of wildtype and Δ*atg4* mutant hyphae, treated with solvent (Control) and azoles for 4 days. Sample size n= 3 independent experiments with 45-123 cells each

(**m,n**) Germination and appressorium formation after 4 - 5 h in the presence of 10 µg ml^{-1} epoxiconazole or metconazole. Sample size n= 4-7 independent experiments with 79-1831 cells each (**n**). Scale bar in (**m**) = 20 µm.

Cells grown in CM-Glucose, 25°C, 100 rpm and treated for 24 h with 10 µg ml^{-1} azoles. Results shown in (**c**) were obtained independently in 3 experiments, (**e**,**j**,**k**) were obtained independently in 2 experiments and (**m**) were obtained independently in 4-7 experiments. Data sets in (**f**,**g,i**) did not pass normality testing (Shapiro-Wilk test, P<0.05) and are given as Whiskers' plots with 25/75 percentiles (blue lines), median (red line) and minimum and maximum (whiskers ends); bars in (**a,b**,**d**,**h**,**l,n**) represent mean ± SEM, dots indicate independent experiments; statistical analysis in (**f**,**g**,**i**) used non-parametric Mann-Whitney testing, in (**b**,**d**,**h**,**l,n**) Student's t-testing with Welch correction; n.s.= non-significant difference at two-tailed P>0.05; *= significant difference to control at two-tailed P= 0.0154 (**b**, Epoxiconazole), 0.0126 (**b**, Metconazole), 0.0187 **d**, Epoxiconazole), 0.0114 (**d**, Metconazole) and 0.0373 (**l**); **= significant difference to control at two-tailed P= 0.0036 (**h**) and 0.0029 (**l**); ***= significant difference to control at two-tailed P= 0.0007 (**h**), 0.0007 (**l**), 0.0002 (**n**, Dormant) and 0.0003 (**n**, Germ tube);****= two-tailed P<0.0001 (**f**, **g**, **i**, **n**).

All data are provided in the Source Data File.

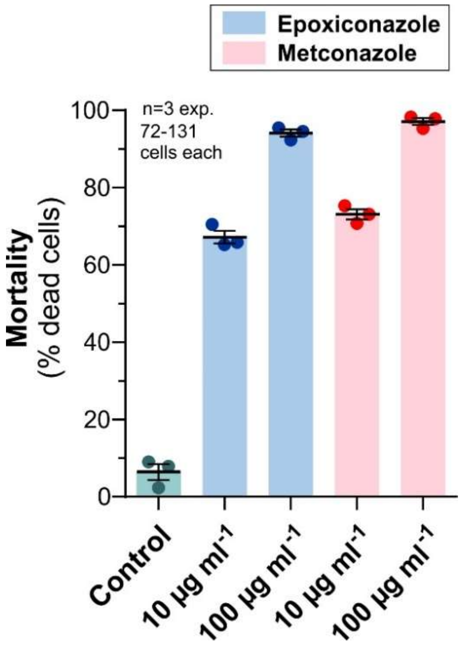

Supplementary Figure 15 Fungicidal activity of azoles in *M. oryzae*.

Hyphae were incubated for 4 days in complete medium, supplemented with 10 µg ml^{-1} and 100 µg ml^{-1} epoxiconazole or metconazole.

Data are given as mean ± standard error of the mean with and sample size n= 3 independent experiments with 72-131 cells each, individual experiments are indicated by red dots. All data are provided in the Source Data File.

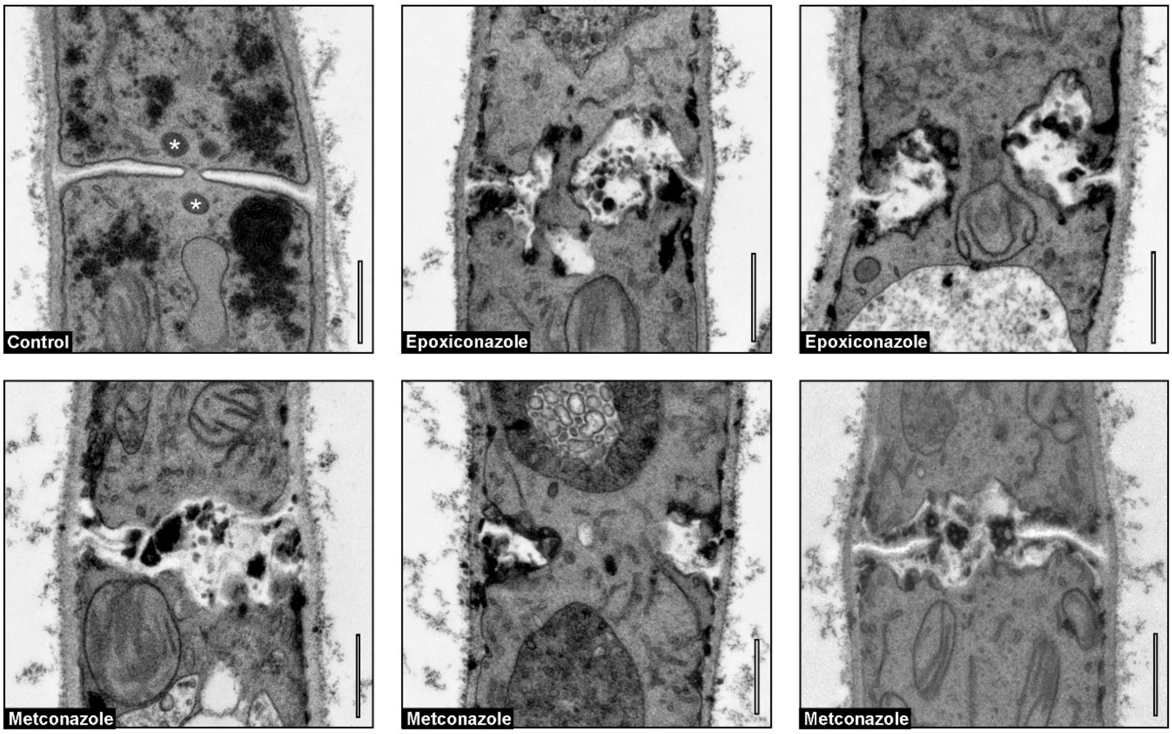

Supplementary Figure 16 Ultrastructure of septae in azole-treated cells of *M. oryzae* hyphae.

Hyphae that were treated with the solvent methanol (Control) contain perforated hyphae, guarded by Woronin bodies (asterisks). 24 h treatment with 10 µg ml^{-1} epoxiconazole or metconazole resulted in aberrant septa. Scale bars= 0.5 µm.

Results shown in were obtained independently in 2 experiments. All data are provided in the Source Data File.

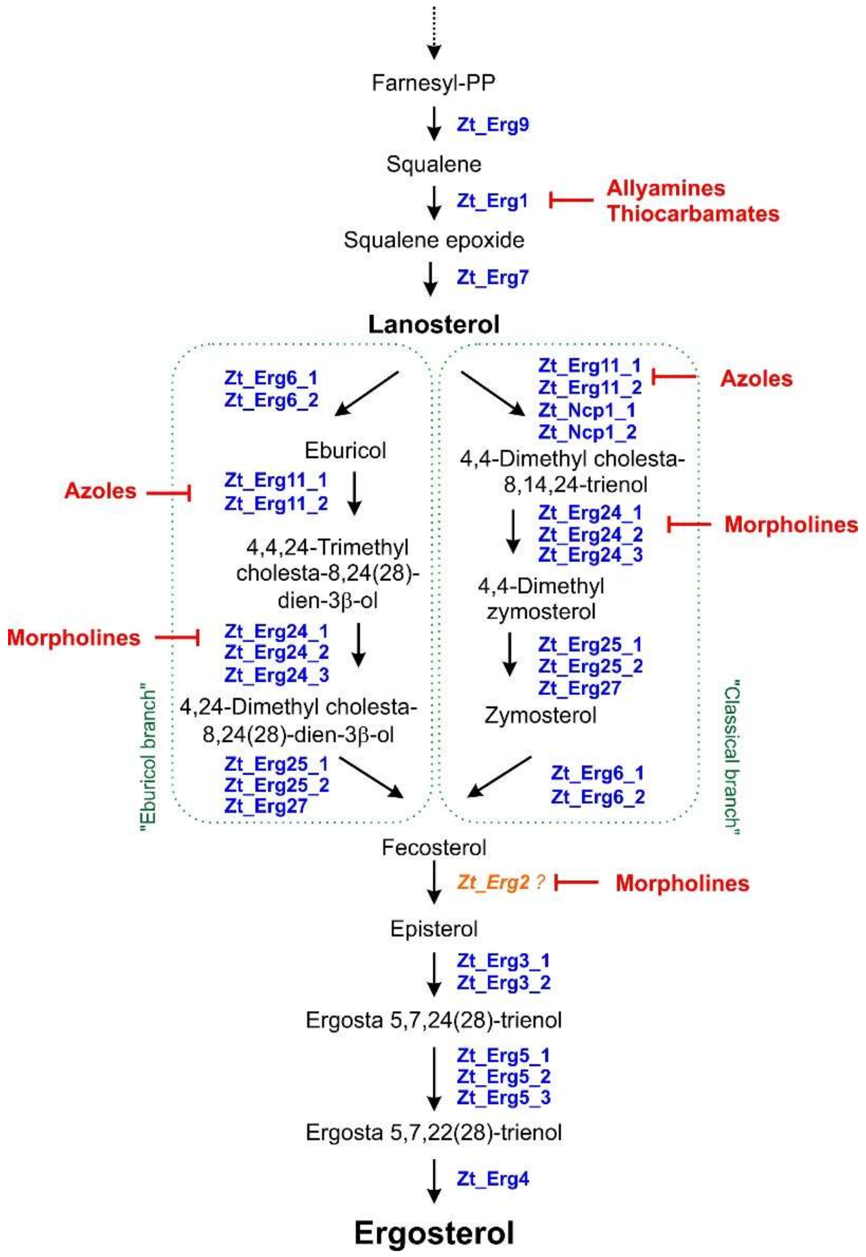

Supplementary Figure 17 Targets of inhibitors of the ergosterol biosynthesis pathway.

UniProt accession numbers of the indicated putative enzymes (blue) in the late ergosterol biosynthesis pathway of *Z. tritici* are: Zt_Erg9, X0L2N3; Zt_Erg_1, X0JFU7; Zt_Erg7, X0K0Z6; Zt_Erg6_1, X0JEW4; Zt_Erg6_2, X0JEM8; Zt_Erg11_1, X0JWG9; Zt_Erg11_2, X0LR56; Zt_Ncp1_1, X0KG25; Zt_Ncp1_2, X0J2V0; Zt_Erg24_1, X0JS28; Zt_Erg24_1, X0K4V7; Zt_Erg24_3, X0INU9; Zt_Erg25_1, X0J3L6; Zt_Erg25_2, X0K4J0; Zt_Erg27, X0L239; Zt_Erg3_1, X0JRF1; Zt_Erg3_2, X0K7Z8; Zt_Erg5_1, X0L0U0; Zt_Erg5_2, X0JBD0; Zt_Erg5_3, X0J6R4; Zt_Erg4,

X0JZ24. Orange: no data base entry for an Erg2 homologue. Note that the "eburicol branch" was demonstrated in *Aspergillus fumigatus*[3], whereas the yeast *Saccharomyces cerevisiae* contains only the "classical branch". No Erg2 homologue was identified in the genome of *Z. tritici* IPO323 (orange).

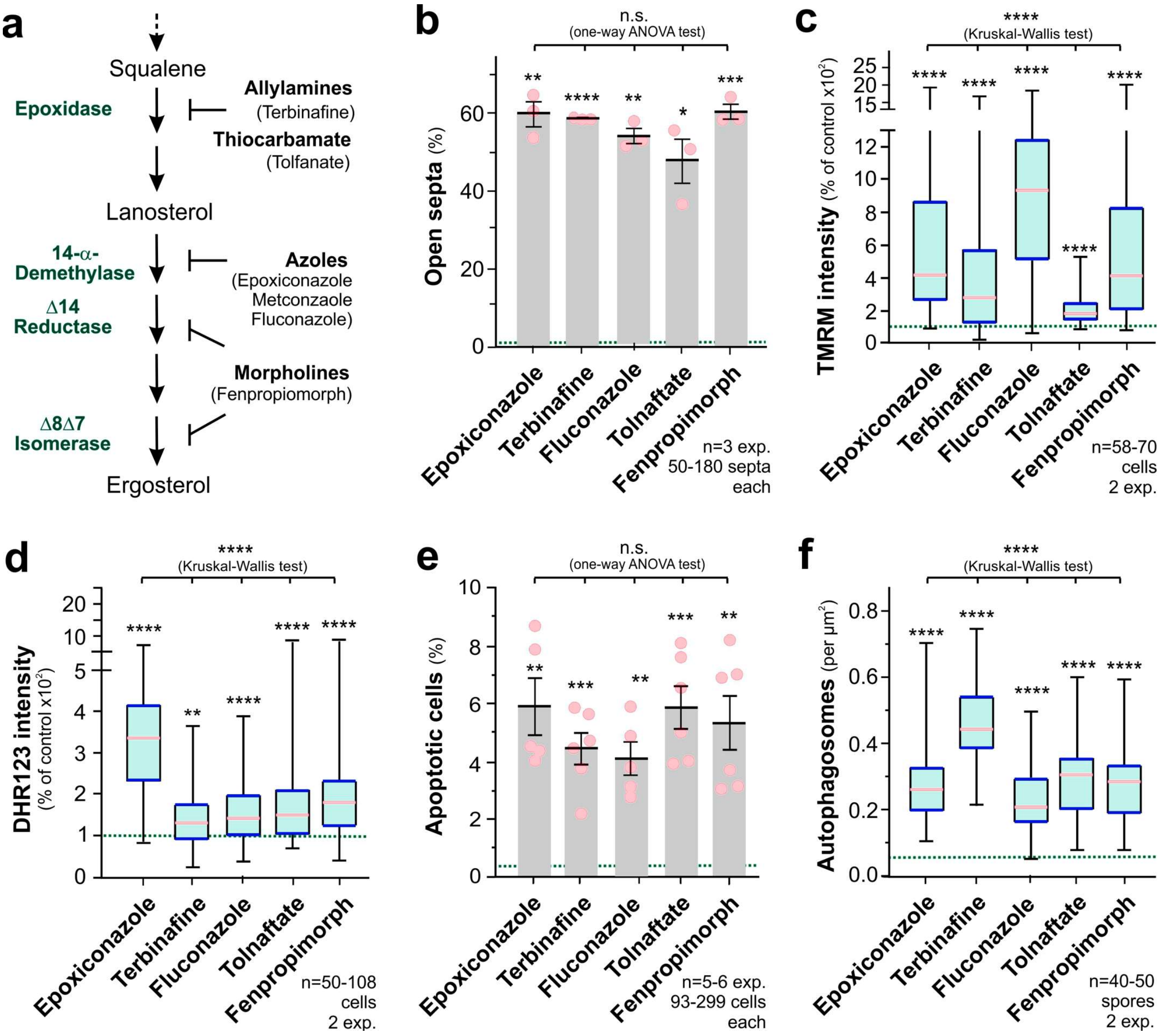

Supplementary Figure 18. Ergosterol biosynthesis inhibitors have a common MoA.

(**a**) Molecular targets of inhibitors of the ergosterol biosynthesis pathway. Enzymes are shown in green; names of inhibitors, used in this study, are provided in parenthesis. See Supplementary Figure 17.

(**b**) Incomplete septation after treatment with ergosterol biosynthesis inhibitors. Sample size n= 3 independent experiments with 50-180 cells each.

(**c**) IMM potential, given as TMRM fluorescence, in cells treated with ergosterol biosynthesis inhibitors. Sample size n= 58-70 cells from 2 independent experiments.

(**d**) mROS, given as DHR-123 fluorescence, in cells treated with ergosterol biosynthesis inhibitors. Sample size n= 50-108 cells from 2 independent experiments.

(**e**) Induction of apoptosis, shown as relative number of FITC-VAD-FMK-stained cells after 24 h, in cells treated with ergosterol biosynthesis inhibitors. Sample size n= 5-6 independent experiments with 93-299 cells each

(**f**) Induction of autophagy, shown as the number eGFP-Atg8-labelled autophagosomes in 1 μm^2 of a maximum projection of a focal plane in cells treated with ergosterol biosynthesis inhibitors. Sample size n= 40-50 spores from 2 independent experiments.

For all experiments cells were grown in YG media at 18°C with 200 rpm and treated for 24 h with 0.01 $\mu g\ ml^{-1}$ (Epoxiconazole), 5 $\mu g\ ml^{-1}$ (Terbinafine), 50 $\mu g\ ml^{-1}$ (Tolfanate), 15 $\mu g\ ml^{-1}$ (Fluconazole)and 5 $\mu g\ ml^{-1}$ (Fenmropiomorph); control experiments contained the corresponding amount of the solvent.

Most data sets in (**c**,**d**,**f**) did not pass a normality test (Shapiro-Wilk test, P<0.05) and are given as Whiskers' plots with 25/75 percentiles (blue lines), median (red line) and minimum and maximum (whiskers ends); bars in (**b**,**e**) represent mean ± SEM; red dots represent independent experiments; statistical comparison with control in (**c**,**d**,**f**) used non-parametric Mann-Whitney testing, and in (**b**,**e**) Student's t-testing with Welch correction; multiple data sets were compared using one-way ANOVA (**b**,**e**) or non-parametric Kruskal-Wallis testing (**c**,**d**,**f**); testing results for comparison to control are: n.s.: non-significant difference at one-side P>0.05; *: two-tailed P values of 0.0140 (**b**); **: two-tailed P values of 0.0026 (**b**, epoxiconazole), 0.0012 (**b,** fluconazole) 0.0045 (**d**), 0.0033 (**e**, epoxiconazole), 0.0021 (**e**, fluconazole) and 0.0029 (**e**, fenpropimorph); ***: two-tailed P values of 0.0009 (**b**), 0.0004 (**e**, terbinafine), and 0.0005 (**e**, tolfonate); ****: two-tailed P values <0.0001 (**b**,**c,d,f**);

testing results for multiple comparisons are: n.s.: non-significant difference at P= 0.0825 (**b**) and 0.6983 (**e**); ****= P value <0.0001 (**c**,**d**,**f**).

All data are provided in the Source Data File.

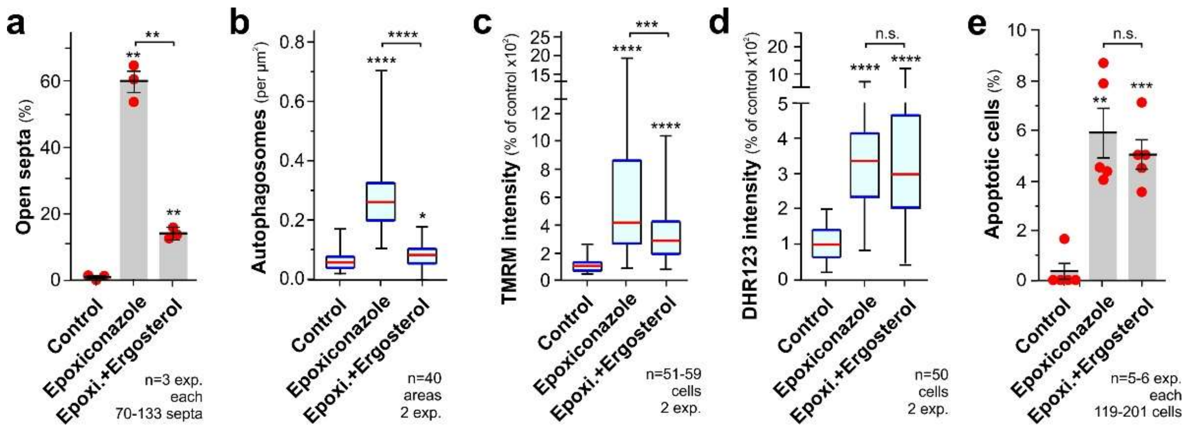

Supplementary Figure 19 Effect of external ergosterol in epoxiconazole-treated *Z. tritici* cells.

(**a**) Number of incomplete septa in cells co-treated with epoxiconazole and exogenous ergosterol.

(**b**) Induction of autophagy, shown as the number eGFP-Atg8-labelled autophagosomes in 1 µm^2 of a maximum projection of a focal plane, in cells co-treated with epoxiconazole and exogenous ergosterol.

(**c**) Mitochondrial inner membrane potential, given as TMRM fluorescence, in cells co-treated with epoxiconazole and exogenous ergosterol.

(**d**) mROS, given as DHR-123 fluorescence, in cells co-treated with epoxiconazole and exogenous ergosterol.

(**e**) Induction of apoptosis, shown as relative number of FITC-VAD-FMK-stained cells after 24h, in cells co-treated with epoxiconazole and exogenous ergosterol.

Cells were grown in YG media at 18°C with 200 rpm and treated for 24 h with 0.01 µg ml^{-1} epoxiconazole and 5 µg ml^{-1} exogenous ergosterol. Most data sets in (**b**,**c**,**d**) did not pass a normality test (Shapiro-Wilk test, P<0.05) and are given as Whiskers' plots (blue lines: 25/75 percentiles; red line: median) with an sample size n= 40 (**b**), n= 51-59 (**c**) and n= 50 (**d**) from 2 independent experiments; bars in (**a,e**) represent

mean ± SEM; sample sizes n= 3 independent experiments (**a**) and n= 5-6 independent experiments (**e**); red dots represent independent experiments; statistical comparison with control in (**b**,**c**,**d**) used non-parametric Mann-Whitney testing, and in (**a**,**e**) Student's t-testing with Welch correction; testing results are: n.s.= non-significant difference at one-side P>0.05; *= significant difference at two-tailed P values of 0.0414 (**b**); **= significant difference at two-tailed P values of 0.0026 (**a**, control compared to epoxiconazole), 0.0016 (**a**, control compared to epoxiconazole + ergosterol), 0.0027 (**a**, epoxiconazole + ergosterol compared to epoxiconazole) and 0.0033 (**e**); ***= significant difference at two-tailed P values of 0.0002 (**c**) and 0.0003 (**e**); ****= significant difference at two-tailed P values of <0.0001 (**b**,**c**,**d**).

All data are provided in the Source Data File.

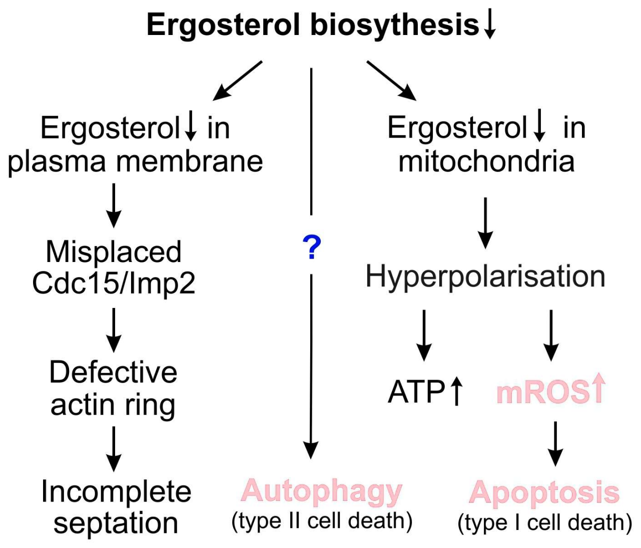

Supplementary Figure 20. The MoA of ergosterol biosynthesis inhibitors

Ergosterol is enriched in the plasma membrane and the inner mitochondrial membrane. Inhibition of ergosterol biosynthesis reduces ergosterol levels in the plasma membrane (PM), which impacts on the formation of actin rings, leading to incomplete septum formation. Inhibition of ergosterol biosynthesis in mitochondria results in hyperpolarization of the IMM, which raises ATP levels in the cytoplasm, but also increases mROS production at respiration complex I. mROS oxidises lipids and

proteins in mitochondria and this is harmful itself. Moreover, increased mROS triggers "suicidal" apoptosis in the pathogen cell (type I programmed cell death). In parallel, changes in the lipid composition of membranes at autophagosome formation sites may induces autophagy (type II programmed cell death). However, other stimuli, such as DNA damage or nutrient shortage due to altered transport over the plasma membrane may trigger autophagy ("?"). Simultaneous inhibition of mROS, apoptosis and autophagy "neutralise" azoles, suggesting that the activation of both programmed cell death pathways, most likely in combination with oxidising mROS underpins the fungicidal activity of azoles in crop pathogens. Note that high azole concentrations induce PM depolarisation *in vitro*.

Supplementary Tables

Supplementary Table 1 Genotype of strains used in this study.

Name	Genotype	Reference
Zymoseptoria tritici		
IPO323_His1ZtG_mChSso1	*MAT1-1* / pGHis1ZtGFP / pHmCherrySSO1, *g418*^{R}, *hyg*^{R},	4
IPO323_eGSso1	*MAT1-1* / pCeGFPSso1*, cbx*^{R}	4
IPO323_mChSsol	*MAT1-1* / pHmCherrySso1, *hyg*^{R}	5
IPO323	Wildtype*, MAT1-1*	6
IPO323_mChSso1	*MAT1-1* / pGmCherrySso1, *g418*^{R}	This study
IPO323_mChSso1_Imp2ZtG	*MAT1-1* / pGmCherrySso1 / pHImp2ZtGFP, *g418*^{R}, *hyg*^{R}	This study
IPO323_LifeactZtG	*MAT1-1* / pCLifeactZtGFP, *cbx*^{R}	4
IPO323_eGAtg8	*MAT1-1* / pCeGFPAtg8, *cbx*^{R}	4
IPO323_ΔAtg4_eGAtg8	*MAT1-1, Δatg4 /* pCeGFPAtg8 *hyg*^{R}*, cbx*^{R}	This study
IPO323_ΔAtg4_ eGSso1	*MAT1-1, Δatg4 /* pCeGFPSso1, *hyg*^{R}*, cbx*^{R}	This study
IPO323_ZtG	*MAT1-1* / pCZtGFP, *cbx*^{R}	7
IPO323_eGAtg8_His1mCh	*MAT1-1* / pCeGFPAtg8 / pGHis1mCherry, *cbx*^{R,} *g418*^{R}	This study
IPO323_mChSso1_P*erg5*ZtG	*MAT1-1* / pHmCherrySso1 / pCP*erg5*ZtGFP, *hyg*^{R}*, cbx*^{R}	This study
IPO323_mChSso1_P*erg6*ZtG	*MAT1-1* / pHmCherrySso1 / pCP*erg6*ZtGFP, *hyg*^{R}*, cbx*^{R}	This study
IPO323_mChSso1_P*sod2*ZtG	*MAT1-1* / pHmCherrySso1 / pCP*sod2*ZtGFP, *hyg*^{R}*, cbx*^{R}	This study
IPO323_mChSso1_P*hyr1*ZtG	*MAT1-1* / pHmCherrySso1 / pCP*hyr1*ZtGFP, *hyg*^{R}*, cbx*^{R}	This study
IPO323_mChSso1_P*mca1*ZtG	*MAT1-1* / pHmCherrySso1 / pCP*mca1*ZtGFP, *hyg*^{R}*, cbx*^{R}	This study
IPO323_mChSso1_P*rny1*ZtG	*MAT1-1* / pHmCherrySso1 / pCP*rny1*ZtGFP, *hyg*^{R}*, cbx*^{R}	This study
IPO323_mChSso1_P*atg1*ZtG	*MAT1-1* / pHmCherrySso1 / pCP*atg1*ZtGFP, *hyg*^{R}*, cbx*^{R}	This study
IPO323_mChSso1_P*atg8*ZtG	*MAT1-1* / pHmCherrySso1 / pCP*atg8*ZtGFP, *hyg*^{R}*, cbx*^{R}	This study
Magnaporthe oryzae		
Guy11	wildtype, *MAT-2*	8
Guy11_Sso1_GFP	*MAT-2*/pPYC1-Sso1-GFP, *sul*^{R}	9
Guy11_GFP-MoATG8	*MAT-2*/pNEB-GFPMoAtg8-BAR, *sul*^{R}	10
Guy11_ΔATG4	*MAT-2, Δatg4, hyg*^{R}	10

P, promoter; *hyg*^{R}, hygromycin resistance; *cbx*^{R}, carboxin resistance; *g418*^{R}, G418 (geneticin) resistance; *sul*^{R}, sulphonylurea resistance *egfp*, enhanced green fluorescent protein; *Ztgfp*, *Z. tritici* codon optimised enhanced green fluorescent protein; *mcherry*, monomeric cherry; *sso*1, a syntaxin-like plasma membrane protein; His, histone; *Imp2* (increased maximal permissive temperature for pim1), F-BAR protein; Lifeact, amino acids 1–17 of actin-binding protein 140 from *S. cerevisiae*, modified for use in filamentous fungi; *erg5*, sterol C-22 desaturase; *erg6*, sterol 24-C-methyltransferase; *sod2*, superoxide dismutase; *hyr1*, glutathione peroxidase; *mca1*, metacaspase; *rny1*, RNase in yeast; *atg1*, serine-threonine protein kinase; Atg8, autophagosome maturation protein,

Supplementary Table 2 Plasmids used in this study.

Name	Description	Reference
Zymoseptoria tritici		
pGHis1ZtGFP	P*tub2-his1-ztgfp*, *g418*^{R}	4
pGmCherrySso1	P*tub2-mCherry-sso1*, *g418*^{R}	4
pCeGFPSso1	P*tub2-egfp-sso1*, *cbx*^{R}	4
pHmCherrySso1	P*tub2-mCherry-sso1*, *hyg*^{R}	4
pHImp2ZtGFP	P*imp2-imp2-ztgfp*, *hyg*^{R}	This study
pChygYR	P*trpC-hph, hyg*^{R}	11
pCLifeactZtGFP	P*tub2-lifeact-ztgfp*, *cbx*^{R}	4
pCeGFPAtg8	P*tub2-egfp-atg8*, *cbx*^{R}	4
pHΔAtg4	Δ*atg4*, *hyg*^{R}	This study
pGHis1mCherry	P*tub2-his1-mCherry*, *g418*^{R}	This study
pCZtGFP	P*tub2-ztgfp, cbx*^{R}	7
pCP*erg5*ZtGFP	P*erg5-ztgfp, cbx*^{R}	This study
pCP*erg6*ZtGFP	P*erg6-ztgfp, cbx*^{R}	This study
pCP*sod2*ZtGFP	P*sod2-ztgfp, cbx*^{R}	This study
pCP*hyr1*ZtGFP	P*hyr1-ztgfp, cbx*^{R}	This study
pCP*mca1*ZtGFP	P*mca1-ztgfp, cbx*^{R}	This study
pCP*rny1*ZtGFP	P*rny1-ztgfp, cbx*^{R}	This study
pCP*atg1*ZtGFP	P*atg1-ztgfp, cbx*^{R}	This study
pCP*atg8*ZtGFP	P*atg8-ztgfp, cbx*^{R}	This study
Magnaporphe oryzae		
pPYC1-Sso1_GFP	P*sso1-sso1-gfp*, *sul*^{R}	9
pNEB-GFPMoAtg8-BAR	P*atg8-gfp-atg8*, *bar*^{R}	10

P, promoter; *hyg*^{R}, hygromycin resistance; *cbx*^{R}, carboxin resistance; *g418*^{R}, G418 (Geneticin) resistance; *sul*^{R}, Sulphonylurea resistance *egfp*, enhanced green fluorescent protein; *Ztgfp*, *Z. tritici* codon optimised enhanced green fluorescent protein; *mcherry*, monomeric cherry; *sso*1, a syntaxin-like plasma membrane protein; *his1*, histone1; *Imp2* (increased maximal permissive temperature for pim1), F-BAR protein; *lifeact*, amino acids 1–17 of actin-binding protein 140 from *S. cerevisiae*, modified for use in filamentous fungi; *erg5*, sterol C-22 desaturase; *erg6*, sterol 24-C-methyltransferase; *sod2*, superoxide dismutase; *hyr1*, glutathione peroxidase; *mca1*, metacaspase; *rny1*, RNase in yeast; *atg1*, serine-threonine protein kinase; *atg8*, autophagosome maturation protein.

Supplementary Table 3 Experimental strain usage.

Strain name	Type of experiment	Figure or Video
IPO323_His1ZtG_mChSso1	Assessment of septa opening	Fig. 1a; Fig.2f; Fig. 4d; Fig. S5c; Video 3
IPO323_eGSso1	Live dead analysis; morphology index and volume analysis; assessment of septa opening; mitochondrial potential measurement	Fig. 1b-g; Fig. 2a-d, 2g; Fig. 3c, 3d; Fig. 5j, 5l-n; Fig.8b, 8c; Fig. S2a-c; Fig. S6h; Fig. S11a, S11b; Video 1; Video 2
IPO323_mChSsoI	Plasma membrane permeability; mROS and Apoptosis analysis	Fig. 1h, 1i; Fig. 4a, 4b,4e-j; Fig. 6g, Fig. 8d, 8e; Fig. S4b, S4c; Fig. S5d, S5e; Fig. S7a, S7b, S7d; Fig. S11c, S11d
IPO323	Ultrastructure analysis of different compartments; ATP analysis	Fig. 2e; Fig. 3a, 3b, 3e; Fig.4c; Fig. 5g; Fig. S1a, S1b; Fig. S4a; Fig. S5a, S5b; Fig. S6c, S6d
IPO323_mChSso1_ Imp2ZtG	Investigation of localisation of septation-associated F-BAR protein	Fig. 2j; Fig. S3a, S3b; Video 4
IPO323_LifeactZtG	Investigation of actin ring formation and morphology	Fig. 2h, 2i; Video 5
IPO323_eGAtg8	Assessment of the azole effect on autophagy	Fig. 5a-e, 5h, 5i, 5k; Fig. 8f; Fig. S6a, S6e-g; Fig.S7c; Fig. S11e; Video 6
IPO323_ΔAtg4_eGAtg8	Assessment of the azole effect on autophagy	Fig. 5h, 5i, 5k; Fig. S6e, S6f
IPO323_ΔAtg4_eGSso1	Live dead analysis	Fig. 5j; Fig. S6i
IPO323_eGAtg8_His1mCh	Co-visualisation of autophagosomes and nucleus	Fig. 5f; Fig. S6b
IPO323_ZtG	Live dead analysis *in planta*	Fig. 6a, 6b
IPO323_mChSso1_P*erg5*ZtG	Reporter for sterol depletion	Fig. 6e, 6f
IPO323_mChSso1_P*erg6*ZtG	Reporter for sterol depletion	Fig. 6e, 6f
IPO323_mChSso1_P*sod2*ZtG	Reporter for mROS	Fig. 6e, 6f
IPO323_mChSso1_P*hyr1*ZtG	Reporter for mROS	Fig. 6e, 6f
IPO323_mChSso1_P*mca1*ZtG	Reporter for apoptosis	Fig. 6c-f
IPO323_mChSso1_P*rny1*ZtG	Reporter for apoptosis	Fig. 6e, 6f
IPO323_mChSso1_P*atg1*ZtG	Reporter for autophagy	Fig. 6e, 6f
IPO323_mChSso1_P*atg8*ZtG	Reporter for autophagy	Fig. 6e, 6f
Guy11	Ultrastructure analysis of different compartments; mROS, Apoptosis and mitochondrial potential measurement	Fig. 7a, 7e-h, 7k-n; Fig. S1c, S1d; Fig. S8; Fig. S9
Guy11_Sso1-GFP	Assessment of septa opening	Fig. 7b-d; Video 7
Guy11_GFP-MoATG8	Assessment of the azole effect on autophagy	Fig. 7i, 7j
Guy11_ΔATG4	Assessment of the azole effect on autophagy	Fig. 7l

Supplementary Table 4 Reporter genes to study the MoA of azoles *in planta*.

Protein	Name	Reporter	Accession numbers		Domains[a]		Identity/ Similarity[b]	Reference[c]
			Z. tritici	*S. cerevisiae*	*Z. tritici*	*S. cerevisiae*		
**Erg5**	Sterol C-22 desaturase	Sterol depletion	XP_003855007.1	NP_013728.1	p450 (4.7e-59)	p450 (3e-55)	49.1/66.7	1
**Erg6**	Sterol 24-C-methyltransfer ase	Sterol depletion	XP_003853943.1	NP_013706.1	Methyltransf_11 (2.8e-22) Sterol_MT_C (6.1e-30)	Methyltransf_11 (4.5e-21) Sterol_MT_C (9e-28)	52.2/67.4	1
**Sod2**	Superoxide dismutase	mROS	XP_003853493.1	AJU21583.1	Sod_Fe_N (4.8e-33) Sod_Fe_C (5.2e-42)	Sod_Fe_N (3.2e-25) Sod_Fe_C (2.6e-34)	33.7/48.4	12, 13
**Hyr1**	Glutathione peroxidase	mROS	XP_003856417.1	EGA61848.1	GSHPx (1.5e-39)	GSHPx (2.5e-51)	52.7/68.6	14
**Mca1**	Metacaspase	Apoptosis	XP_003848535.1	EDN63533.1	Peptidase_C14 (8.5e-46)	Peptidase_C14 (2.9e-47)	50.5/59.5	15
**Rny1**	RNase in yeast	Apoptosis	XP_003848637.1	QHB12138.1	Ribonuclease_T (2.3e-45)	Ribonuclease_T (2.9e-45)	30.6/44.3	16
**Atg1**	Serine-threonine protein kinase	Autophagy	XP_003850609.1	QHB08479.1	Pkinase (1.3e-61) ATG1-like_MIT1 (2.9e-47) ATG1-like_MIT2 (1.1e-29)	Pkinase (2e-60) ATG1-like_MIT1 (7.1e-44) ATG1-like_MIT2 (8.1e-30)	28.4/44.5	17
**Atg8**	Ubiquitin-like protein	Autophagy	XP_003855091.1	QHB06698.1	ATG8 (2.5e-51)	ATG8 (1.1e-50)	77.3/88.2	17

[a]Determined in PfamScan (https://www.ebi.ac.uk/Tools/pfa/pfamscan/), error probability in brackets.
[b]Given in percentage; determined in EMBOSS Needle (http://www.ebi.ac.uk/Tools/psa/emboss_needle/).
[c]Reference reporting the identity of the reporter protein in fungi.

Supplementary Table 5 Primers used in this study.

Primer name	Sequence (5' to 3')
SK-Sep-46	GAAGTCTGCGGCAGCTCGCAC
SK-Sep-90	*CCACAAGATCCTGTCCTCGTCCGTCGTCGC*TTACTTGTACAGCTCGTCCATGCCG
SK-Sep-136	CCCAACTGATATTGAAGGAGCATT
SK-Sep-137	CCCGATCTAGTAACATAGATGACA
SK-Sep-215	ATGGTGAGCAAGGGCGAGGAG
SK-Sep-743	*TGGCAGGATATATTGTGGTGTAAACAAATT*TCTCTTCATCTTCATCGTCGTCG
SK-Sep-744	*CCAAAAAAATGCTCCTTCAATATCAGTTGGG*TATGTTCCCATGGAGTATGAGGGA
SK-Sep-745	*GCGCGGTGTCATCTATGTTACTAGATCGGG*TGAGGAAATTGGCGTTTGGGCTAT
SK-Sep-746	*TAAACGCTCTTTTCTCTTAGGTTTACCCGC*TAAGCGCCTCGATCTCTCACAAG
SK-Sep-776	*CATTTGCGGCTGTCTCGAAATCGACGGAAG*AGTCTTCTCAGAGCTTCCTGGAG
SK-Sep-777	*GGTGAAGAGCTCCTCGCCCTTGGAGACCAT*GATGGCGGTAGTGTTGTTGTGATTG
SK-Sep-780	*CATTTGCGGCTGTCTCGAAATCGACGGAAG*ACCCGCTTCTACTGTCAAAAGAGC
SK-Sep-781	*GGTGAAGAGCTCCTCGCCCTTGGAGACCAT*GTTGGCGATGTGTGTTGTGGAGG
SK-Sep-786	*CATTTGCGGCTGTCTCGAAATCGACGGAAG*ATGGAGGCGCAAAGCTAGTGGAC
SK-Sep-787	*GGTGAAGAGCTCCTCGCCCTTGGAGACCAT*CTTGGCTGGTTTTGTCTTGTCTTG
SK-Sep-788	*CATTTGCGGCTGTCTCGAAATCGACGGAAG*ACATGATCTACACATCTTCCCATATAT
SK-Sep-789	*GGTGAAGAGCTCCTCGCCCTTGGAGACCAT*GGACCGTACGGAAGGCATGGTGA
SK-Sep-802	*CATTTGCGGCTGTCTCGAAATCGACGGAAG*GATTATACGTCGTTGGTACCATCC
SK-Sep-803	*GGTGAAGAGCTCCTCGCCCTTGGAGACCAT*GATGACAGCGATGGTGCAAGTGC
SK-Sep-804	*CATTTGCGGCTGTCTCGAAATCGACGGAAG*TGAAGCCGCGCGTGCGCCAAT
SK-Sep-805	*GGTGAAGAGCTCCTCGCCCTTGGAGACCAT*GGCGGCGATTCGTGTTTGGTTG
SK-Sep-808	*CATTTGCGGCTGTCTCGAAATCGACGGAAG*GATGCGAAGGAATGCTCGTCAAC
SK-Sep-809	*GGTGAAGAGCTCCTCGCCCTTGGAGACCAT*GTTTGAGGATGAAGCACGGCCAA
SK-Sep-810	*CATTTGCGGCTGTCTCGAAATCGACGGAAG*CCCTTCCAGCCTGCGCGTTCG
SK-Sep-811	*GGTGAAGAGCTCCTCGCCCTTGGAGACCAT*GGTGGAGGAGGAGTTCTTTGGTC
SK-Sep-942	*TGGCAGGATATATTGTGGTGTAAACAAATTTTAGGT*ATGCGCGAATCGCGGG
SK-Sep-943	*GGTGAAGAGCTCCTCGCCCTTGGAGACCATACAC*GCCTTGAGATAATTACTGGG
SK-Sep-949	*CATGTTATCCTCCTCGCCCTTGCTCACCAT*TGCCTTCTTGGGAGTGGCGGC

Italics indicate sequence complementary with another DNA fragment, which allows homologous recombination in *S. cerevisiae*.

Supplementary References

1 Cannon, S. *et al.* Multi-site fungicides suppress banana Panama disease, caused by *Fusarium oxysporum* f. sp. *cubense* Tropical Race 4. *PLoS Pathog* **18**, e1010860 (2022).

2 Ren, L. *et al.* The Cdc15 and Imp2 SH3 domains cooperatively scaffold a network of proteins that redundantly ensure efficient cell division in fission yeast. *Mol. Biol. Cell* **26**, 256-269 (2015).

3 Alcazar-Fuoli, L. *et al.* Ergosterol biosynthesis pathway in *Aspergillus fumigatus*. *Steroids* **73**, 339-347 (2008).

4. Kilaru, S., Schuster, M., Ma, W. & Steinberg, G. Fluorescent markers of various organelles in the wheat pathogen *Zymoseptoria tritici*. *Fungal Genet. Biol.* **105**, 16-27 (2017).

5 Steinberg, G. *et al.* A lipophilic cation protects crops against fungal pathogens by multiple modes of action. *Nat. Commun.* **11**, 1608 (2020).

6 Kema, G. H. & van Silfhout, C. H. Genetic variation for virulence and resistance in the wheat-*Mycosphaerella graminicola* pathosystem III. Comparative seedling and adult plant experiments. *Phytopathol.* **87**, 266–272 (1997).

7 Kilaru, S. *et al.* A codon-optimized green fluorescent protein for live cell imaging in *Zymoseptoria tritici*. *Fungal Genet. Biol.* **79**, 125-131 (2015).

8 Leung, H., Borromeo, E. & Notteghem, J. L. Genetic analysis of virulence in the rice blast fungus *Magnaporthe grisea*. *Phytopathol.* **78**, 1227-1233 (1988).

9 Giraldo, M. C. *et al.* Two distinct secretion systems facilitate tissue invasion by the rice blast fungus *Magnaporthe oryzae*. *Nat. Commun.* **4**, 1996 (2013).

10 Kershaw, M. J. & Talbot, N. J. Genome-wide functional analysis reveals that infection-associated fungal autophagy is necessary for rice blast disease. *Proc. Natl. Acad. Sci.U S A* **106**, 15967-15972 (2009).

11 Kilaru, S. & Steinberg, G. Yeast recombination-based cloning as an efficient way of constructing vectors for *Zymoseptoria tritici*. *Fungal Genet. Biol.* **79**, 76-83 (2015).

12 Gralla, E. B. & Kosman, D. J. Molecular genetics of superoxide dismutases in yeasts and related fungi. *Adv. Genet.* **30**, 251-319 (1992).

13 Fabrizio, P. *et al.* SOD2 functions downstream of Sch9 to extend longevity in yeast. *Genetics* **163**, 35-46 (2003).

14 Huang, K., Czymmek, K. J., Caplan, J. L., Sweigard, J .A. & Donofrio, N. M. HYR1-mediated detoxification of reactive oxygen species is required for full virulence in the rice blast fungus. *PLoS Pathog* **7**, e1001335 (2011).

15 Madeo, F. *et al.* A caspase-related protease regulates apoptosis in yeast. *Mol. Cell* **9**, 911-917 (2002).

16 Thompson, D. M. & Parker, R. The RNase Rny1p cleaves tRNAs and promotes cell death during oxidative stress in *Saccharomyces cerevisiae*. *J. Cell Biol.* **185**, 43-50 (2009).

17 Bernard, A., Jin, M., Xu, Z. & Klionsky, D. J. A large-scale analysis of autophagy-related gene expression identifies new regulators of autophagy. *Autophagy* **11**, 2114-2122 (2015).
