# Supplementary material for: Azoles activate type I and type II programmed cell death pathways in crop pathogenic fungi
Source: Nat Commun. 2024 May 31;15:4357. doi: 10.1038/s41467-024-48157-9 (PMC11143370; doi:10.1038/s41467-024-48157-9)
Supplement: Supplementary file 3 — Description of Additional Supplementary Files [file 41467_2024_48157_MOESM3_ESM.pdf]

Description of Additional Supplementary Files:

Supplementary Movie 1: Fluorescent recovery of the plasma membrane syntaxin eGFP-Sso1 in solvent- and epoxiconazole-treated cells. After the plasma membrane was photobleached with a 405 nm laser pulse, fluorescence recovers due to lateral diffusion of eGFP-Sso1 into the darkened area. This recovery is impaired in epoxiconazole-treated cells (0.01 µg ml-1 epoxiconazole for 24 h). Time is given in seconds:milliseconds. Scale bar= 2 µm.

Supplementary Movie 2: Incomplete septation in azole-treated Z. tritici spores. 3D reconstruction of septa, visualised by the plasma membrane syntaxin eGFP-ZtSso1, in cells, treated for 24 h with the solvent methanol (0.1% v v-1 , Control), 0.01 µg ml-1 epoxiconazole or 0.01 µg ml-1 metconazole. Scale bar= 3 µm.

Supplementary Movie 3: Laser-rupture experiment in control and epoxiconazole-treated Z. tritici spores. The tip cell of multi-cellular spores (yellow arrowhead) was disrupted by a laser pulse. Control cells concealed the damage by "plugging" their septal pores with Woronin bodies, indicated by the stationary nuclei (green). epoxiconazoletreated cells cannot close the incomplete septa, which results in leaking of nuclei from subapical cells towards the wounding. The plasma membrane is visualised by mCherryZtSso1 (red) and nuclei by His1-ZtGFP (green). Time is given in seconds:milliseconds. Scale bar= 5 µm.

Supplementary Movie 4: Effect of epoxiconazole treatment on localisation of a putative F-BAR protein ZtImp2. In solvent-treated cells (Control), the Cdc15-family protein ZtImp2-ZtGFP localises to the growing septa. Treatment with epoxiconazole results prevents localization of the putative F-BAR protein at septae. Scale bar= 2 µm.

Supplementary Movie 5: Incomplete F-actin rings in azole-treated Z. tritici spores. 3D reconstruction of an open actin ring in methanol-treated spore (Control), and incomplete F-actin rings in cells, treated for 24 h with 0.01 µg ml-1 epoxiconazole or metconazole. F-actin was visualised with Lifeact-ZtGFP. Scale bar= 2 µm.

Supplementary Movie 6: Phagophores in epoxiconazole-treated Z. tritici spores. 3D reconstruction of large autophagosomes in living cells, treated for 24 h with 0.01 µg ml-1 epoxiconazole. The organelles were visualised with eGFP-Atg8. Scale bar= 2 µm.

Supplementary Movie 7: Incomplete septation in azole-treated M. oryzae hyphae. 3D reconstruction of septa, visualised by the plasma membrane syntaxin Sso1-eGFP, in M. oryzae hyphal cells, treated for 24 h with the solvent methanol (0.1% v v-1 , Control), 10 µg ml-1 epoxiconazole. Scale bar= 3 µm.
