# Supplementary figures and images for: Azoles activate type I and type II programmed cell death pathways in crop pathogenic fungi
Source: Nat Commun. 2024 May 31;15:4357. doi: 10.1038/s41467-024-48157-9 (PMC11143370; doi:10.1038/s41467-024-48157-9)

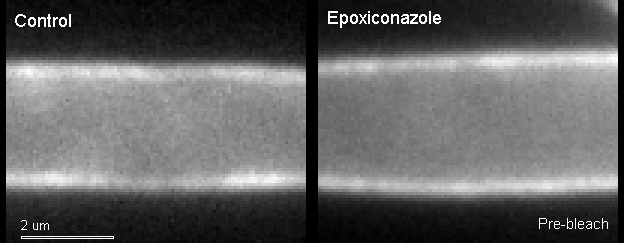

Supplement: Supplementary file 4 — Supplementary Movie 1 [file 41467_2024_48157_MOESM4_ESM.gif]

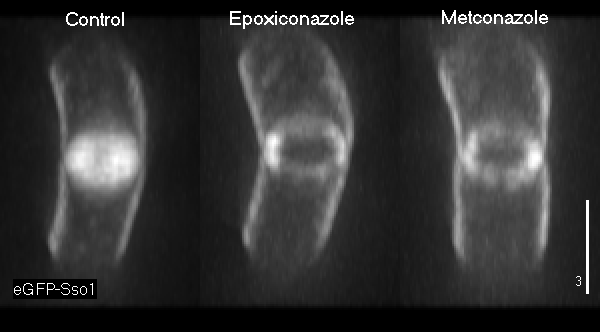

Supplement: Supplementary file 5 — Supplementary Movie 2 [file 41467_2024_48157_MOESM5_ESM.gif]

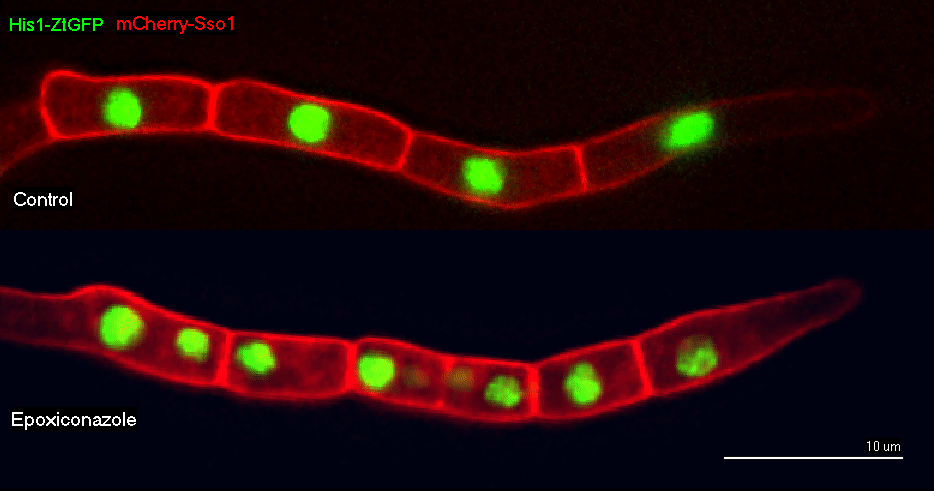

Supplement: Supplementary file 6 — Supplementary Movie 3 [file 41467_2024_48157_MOESM6_ESM.gif]

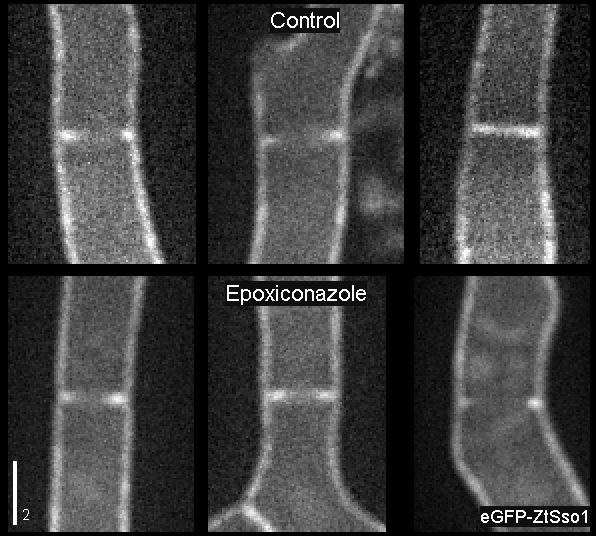

Supplement: Supplementary file 7 — Supplementary Movie 4 [file 41467_2024_48157_MOESM7_ESM.gif]

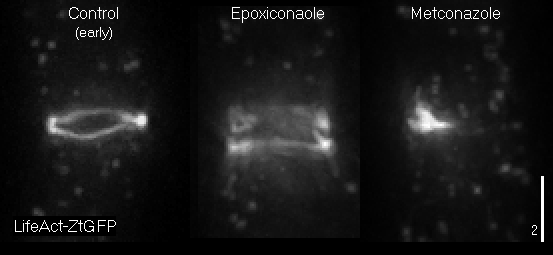

Supplement: Supplementary file 8 — Supplementary Movie 5 [file 41467_2024_48157_MOESM8_ESM.gif]

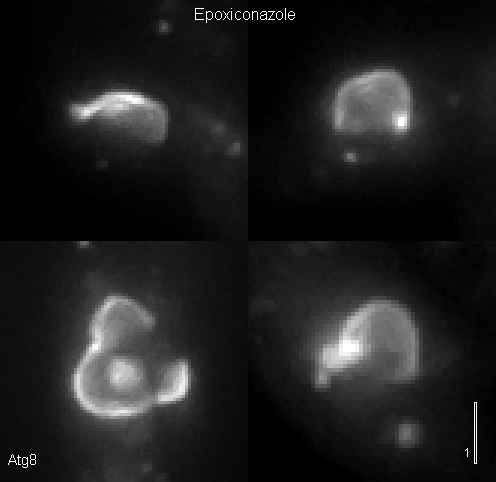

Supplement: Supplementary file 9 — Supplementary Movie 6 [file 41467_2024_48157_MOESM9_ESM.gif]

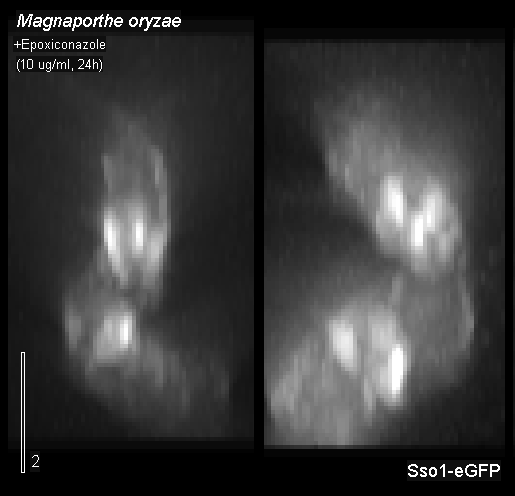

Supplement: Supplementary file 10 — Supplementary Movie 7 [file 41467_2024_48157_MOESM10_ESM.gif]
